# Ubiquitylation activates a peptidase that promotes cleavage and destabilization of its activating E3 ligases and diverse growth regulatory proteins to limit cell proliferation in *Arabidopsis*

**DOI:** 10.1101/gad.292235.116

**Published:** 2017-01-15

**Authors:** Hui Dong, Jack Dumenil, Fu-Hao Lu, Li Na, Hannes Vanhaeren, Christin Naumann, Maria Klecker, Rachel Prior, Caroline Smith, Neil McKenzie, Gerhard Saalbach, Liangliang Chen, Tian Xia, Nathalie Gonzalez, Mathilde Seguela, Dirk Inzé, Nico Dissmeyer, Yunhai Li, Michael W. Bevan

**Affiliations:** 1 John Innes Centre, Norwich NR4 7QA, United Kingdom;; 2 State Key Laboratory of Plant Cell and Chromosome Engineering, CAS Centre of Excellence in Molecular Plant Biology, Institute of Genetics and Developmental Biology, Chinese Academy of Sciences, Beijing 100101, China;; 3 VIB-UGent Centre for Plant Systems Biology, Ghent University, 9052 Gent, Belgium;; 4 Leibniz Institute of Plant Biochemistry (IPB), D-06120 Halle, Germany

**Keywords:** ubiquitylation, organ size, *Arabidopsis*, ubiquitin-activated peptidase, N-end rule-mediated degradation

## Abstract

The characteristic shapes and sizes of organs are established by cell proliferation patterns and final cell sizes, but the underlying molecular mechanisms coordinating these are poorly understood. Here we characterize a ubiquitin-activated peptidase called DA1 that limits the duration of cell proliferation during organ growth in *Arabidopsis thaliana*. The peptidase is activated by two RING E3 ligases, Big Brother (BB) and DA2, which are subsequently cleaved by the activated peptidase and destabilized. In the case of BB, cleavage leads to destabilization by the RING E3 ligase PROTEOLYSIS 1 (PRT1) of the N-end rule pathway. DA1 peptidase activity also cleaves the deubiquitylase UBP15, which promotes cell proliferation, and the transcription factors TEOSINTE BRANCED 1/CYCLOIDEA/PCF 15 (TCP15) and TCP22, which promote cell proliferation and repress endoreduplication. We propose that DA1 peptidase activity regulates the duration of cell proliferation and the transition to endoreduplication and differentiation during organ formation in plants by coordinating the destabilization of regulatory proteins.

## Abstract (precis)

In this study, Dong et al. investigated the molecular mechanisms coordinating characteristic shapes and sizes of organs in plants. The authors characterize a ubiquitin-activated peptidase called DA1, which regulates the duration of cell proliferation and the transition to endoreduplication and differentiation during organ formation in plants by coordinating the destabilization of regulatory proteins.

The shapes and sizes of organs are established by mechanisms that orient cell proliferation and determine the final numbers and sizes of cells forming the organ. Transplantation experiments showed that some animal organs have an intrinsic mechanism that determines their final size by controlling the duration of cell proliferation (Barry and Camargo 2013), which is controlled in part by the HIPPO/YAP pathway that limits cell proliferation and promotes apoptosis (Pan 2010). However, the mechanisms coordinating cell proliferation and cell size during organ growth remain poorly understood (Johnston and Gallant 2002). Due to the simpler planar structures of their organs, such as leaves and petals, and the absence of cell movement due to rigid cell walls, plants have some experimental advantages for studying organ growth (Green et al. 2010).

Leaf growth in plants is initiated at shoot meristems (for review, see Sluis and Hake 2015). After specification of boundaries and growth axes, the leaf lamina grows in an initial period of cell division in which cell size is relatively constant, followed by a transition to endoreduplication associated with cell expansion and differentiation (Breuer et al. 2010; De Veylder et al. 2011). The transition from cell proliferation to cell expansion is spatially and temporarily regulated during leaf growth and appears to progress from the tip to the base of the leaf as a cell division arrest front (Kazama et al. 2010) accompanied by shifts in gene expression patterns (Efroni et al. 2008; Andriankaja et al. 2012). A key question is how the transition from cell proliferation to cell expansion and differentiation is coordinated to generate a correctly sized organ.

The RING E3 ligases Big Brother (BB) (Disch et al. 2006) and DA2 (Xia et al. 2013) limit the duration of cell proliferation during organ growth. Members of the *DA1* family also limit cell proliferation (Li et al. 2008), and loss-of-function mutations in *BB* and *DA2* interact synergistically with the *da1-1* allele of *DA1* to increase organ and seed size in *Arabidopsis* (Li et al. 2008; Xia et al. 2013), suggesting that one of their growth-limiting activities is mediated by enhancing the growth-repressive activity of DA1 family members. Genetic analyses showed that DA1 reduced the stability of both UBP15 (Du et al. 2014), a deubiquitylation enzyme promoting cell proliferation (Liu et al. 2008), and TEOSINTE BRANCED 1/CYCLOIDEA/PCF 14 (TCP14) and TCP15 proteins (Peng et al. 2015), which repress endoreduplication by transcriptional control of *RETINOBLASTOMA-RELATED1* (*RBR1*) and *CYCLIN A2;3* (*CYCA2;3*) gene expression (Li et al. 2012).

Here we show that DA1 is an endopeptidase activated by multiple ubiquitylations mediated by the E3 ligases BB and DA2. In a feedback mechanism, DA1 then cleaves BB and DA2, leading to their destabilization. DA1-mediated cleavage of BB exposed a destabilizing N-terminal that was substrate for the N-end rule E3 ligase PROTEOLYSIS 1 (PRT1). This mechanism is predicted to transiently activate DA1 peptidase, which also cleaves UBP15, TCP15, and the related TCP22, leading to their predicted inactivation and destabilization. DA1 peptidase may therefore contribute to the concerted transition from cell proliferation to endoreduplication and differentiation, limiting organ size.

## Results

### Genetic and physical interactions of DA1, BB, and DA2

We previously identified genetic interactions between the *da1-1* allele of *DA1* and genes encoding the RING E3 ligases BB (Li et al. 2008) and DA2 (Xia et al. 2013) that led to synergistic increases in seed and organ sizes. In this study, we used the *da1-1*-enhancing allele of *BB* called *eod1-2* (Li et al. 2008) and refer to the mutant version as *bb-eod1-2* and the wild-type version as *BB*. The *da1-1* allele, an R358K change in a highly conserved region, had a negative influence on the functions of *DA1* and the close family member *DAR1*, but the basis of this was not known, which complicated interpretation of DA1 function. We therefore assessed phenotypes of a loss-of function T-DNA allele of *DA1* (*da1-ko1*).

**Figure 1.**
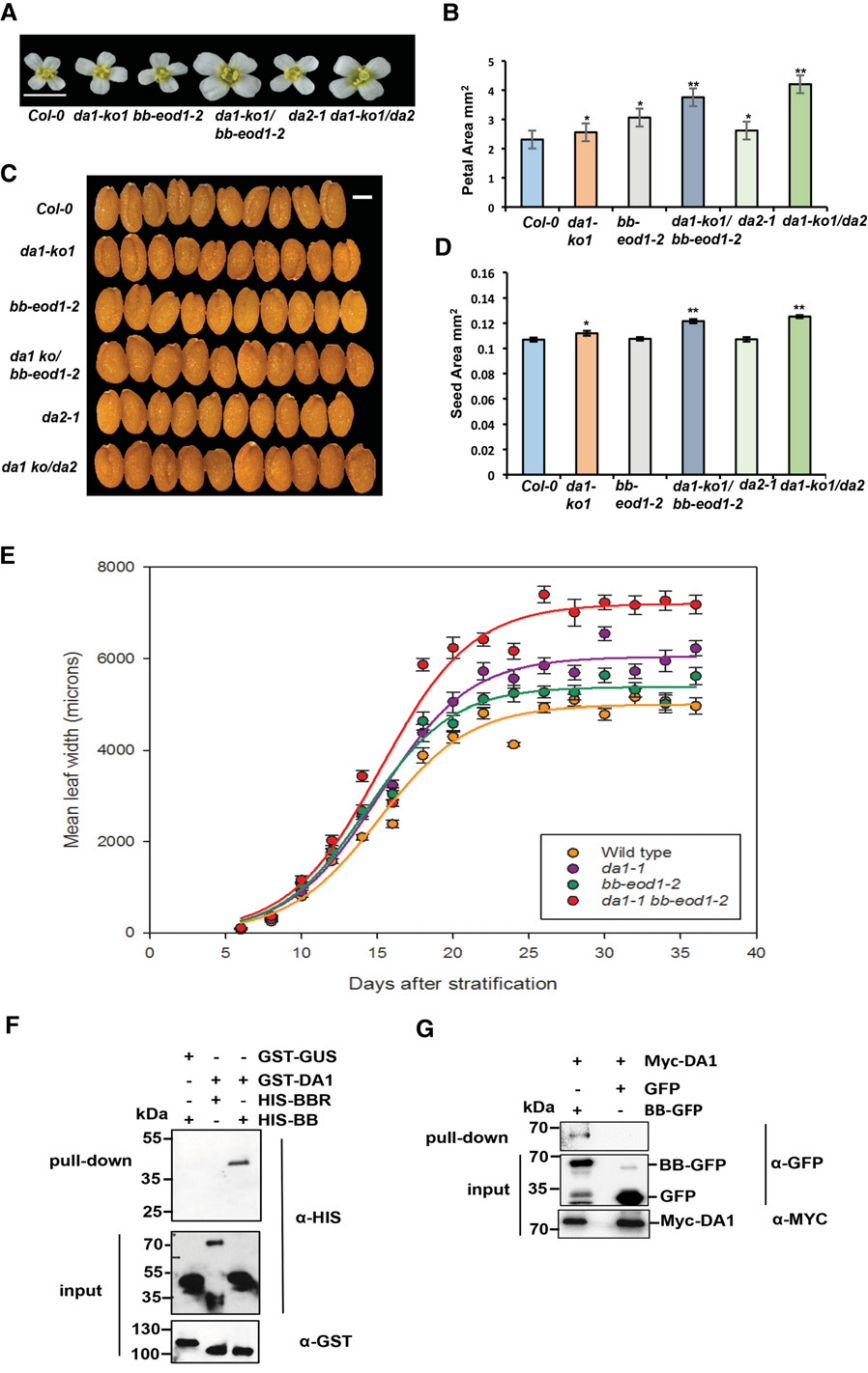
Genetic and physical interactions of DA1, BB, and DA2. (*A*,*B*) The single loss-of-function *da1-ko1* allele interacts with *bb-eod1-2* and *da2-1* to increase petal area. (*A*) An image of flower heads showing the sizes of petals. Bar, 5 mm. (*B*) Petal areas. The values given are means (*n* = 36) ± SE. (*) *P* < 0.05; (**) *P* < 0.01 (Student's *t*-test) compared with wild-type Col-0. (*C*,*D*) The single loss-of-function *da1-ko1* allele interacts with *bb*-*eod1-2* and *da2-1* to increase seed area. (*C*) Ten seeds aligned to reveal size differences. Bar, 2 mm. (*D*) Seed areas. The values given are means (*n* = 50) ± SE. (*) *P* < 0.05; (**) *P* < 0.01 (Student's *t*-test) compared with wild-type Col-0. (*E*) Dynamic growth measurements of leaf 1 width in Col-0, *da1-1*, and *da1-1 bb-eod1-2*. Lines were fitted to data points using the sigmoidal function of Sigmaplot 13. (*F*) DA1 interacts with BB in vitro. GST-DA1 interacted with HIS-BB. GST-DA1 did not interact with HIS-BBR, an E3 ligase closely related to BB. GST-GUS (β-glucuronidase) was used as a negative control. (*G*) Myc-tagged DA1 interacted with BB-GFP after transient coexpression in *N. benthamiana* leaves. BB-GFP and GFP were coexpressed with Myc-DA1 using *Agrobacterium*-mediated transient expression in *N. benthamiana* leaves. Expressed proteins were purified using GFP trap and immunoblotted.

Measurements of petal and seed sizes using high-resolution scanning showed that the *da1-ko1* T-DNA allele led to increased petal (Fig. 1A,B) and seed (Fig. 1C,D) sizes and that it also interacted genetically with the loss-of-function allele *bb-eod1-2* and *da2-1* in both petal size and seed area. This showed that *DA1* can be studied independently of other *DA1* family members. Both the *da1-1* and *bb-eod1-2* mutations increased the maximum growth rate, while the double mutant *da1-1 bb-eod1-2* showed a further increased maximum growth rate and continued to grow for ∼5 d longer than either single mutant (Fig. 1E). The time at maximum growth rates was slightly earlier in *bb-eod1-2* than in Columbia (Col-0), in contrast to *da1-1* and *da1-1 bb-eod1-2*, which showed a 3-d retardation of the time of maximum growth rate, and final leaf sizes showed a more than additive increase in the double mutant, as observed previously (Li et al. 2008). These data indicated that *BB* may influence leaf final size at earlier stages of growth than *DA1*. We demonstrated previously that DA1 and DA2 physically interact (Xia et al. 2013). Pull-down experiments showed that GST-tagged DA1 also interacted with HIS-tagged BB but not with HIS-tagged BBR (BB-related; At3g19910), a close homolog of BB (Fig. 1F; Breuninger and Lenhard 2012). These in vitro interactions were verified by *Agrobacterium*-mediated coexpression of BB-GFP and Myc-tagged DA1 in *Nicotiana benthamiana* leaves. Myc-DA1 was detected only in a complex with BB-GFP and not GFP (Fig. 1G).

### DA1 is multiply ubiquitylated by BB and DA2

**Figure 2.**
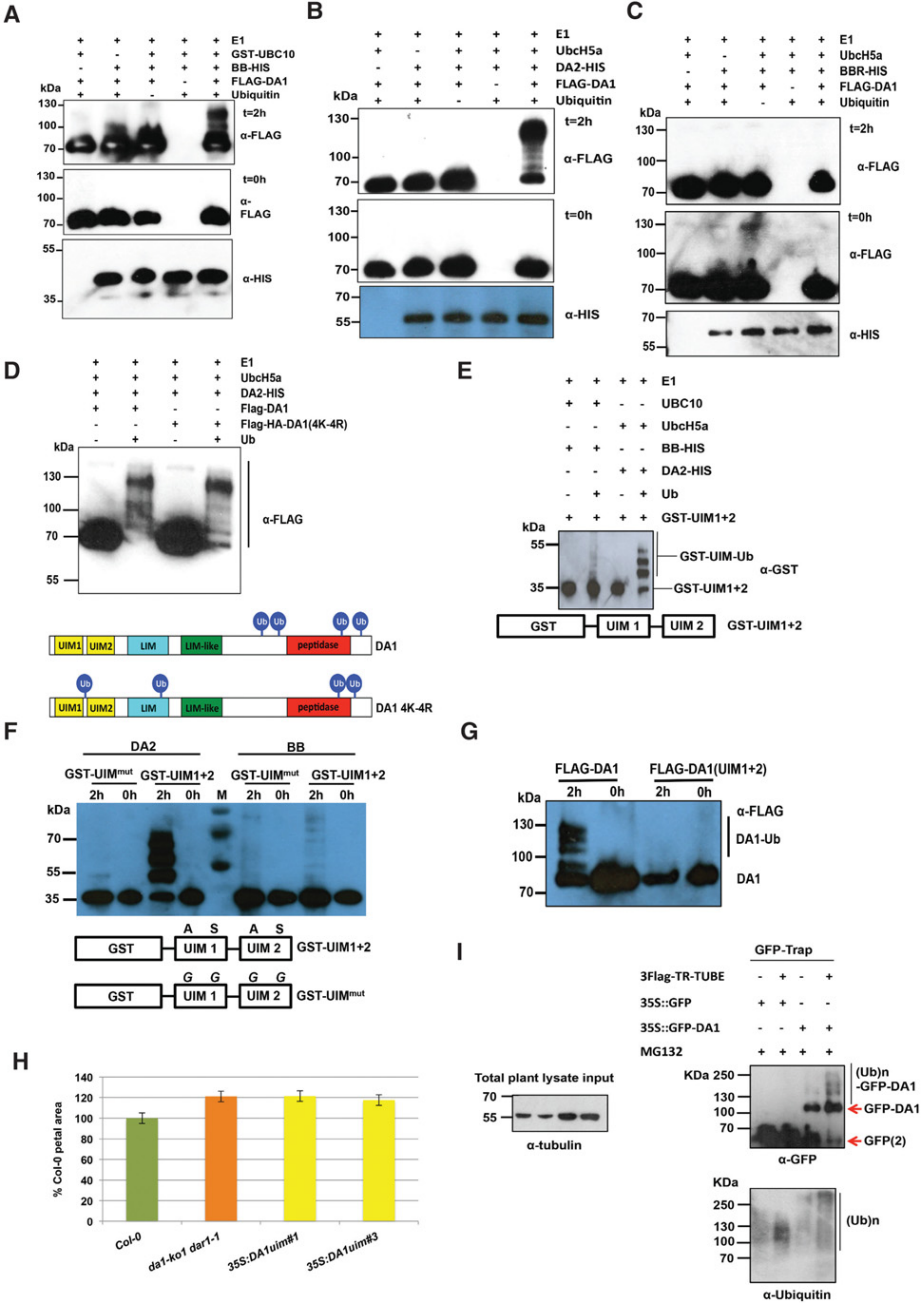
DA1 is multiply ubiquitylated by BB and DA2 in a ubiquitin interaction motif (UIM)-dependent reaction. (*A*–*C*) In vitro ubiquitylation of DA1 by the RING E3 ligases BB (*A*) and DA2 (*B*), but not BBR (*C*), in an E1-, E2-, and ubiquitin-dependent reaction. Anti-Flag antibodies detected Flag-ubiquitylated forms of Flag-DA1 ranging from >70 kDa to ∼130 kDa. Anti-HIS antibodies detected BB-HIS, DA2-HIS, or BBR-HIS fusion proteins. (*D*) Both Flag-DA1 and Flag-DA1(4K-4R) are ubiquitylated by DA2 in similar patterns in an in vitro ubiquitylation reaction. In the *bottom* panel, Ub represents a ubiquitin moiety conjugated to a lysine at the approximate location in DA1 and DA1(4K-4R). Regions of protein similarity with known domains are shown: UIM1 and UIM2 are similar to UIMs, LIM is similar to canonical LIM domains, LIM-like is a related motif found in DA1 family members, and peptidase contains a predicted peptidase active site. (*E*) An in vitro ubiquitylation reaction with DA2 and BB as E3 ligases and GST-UIM1+2. GST-UIM1+2 is ubiquitylated in a pattern similar to that of DA1 by both DA2 and BB, with DA2 conferring higher levels of ubiquitylation than BB. (*F*) An in vitro ubiquitylation reaction with DA2 and BB as E3 ligases and GST-UIM1+2 with mutations that reduce ubiquitin binding. Mutated versions of UIM1 and UIM2 strongly reduced DA2- and BB-mediated ubiquitylation of GST-UIM1+2. (*G*) A time course of Flag-DA1 and Flag-DA1(UIM1+2) with mutations in the UIMs as in *E*. These strongly reduced DA1 ubiquitylation. (*H*) DA1(UIM1+2) is not functional in vivo, as it does not complement the large petal phenotype of the *da1-ko1 dar1-1* double mutant. Two independent homozygous T-DNA insertion lines were scored for petal size and compared with wild-type Col-0 and *da1-ko1 dar1-1*. The values given are means (*n* = 120) ± SE, expressed as the percentage of wild-type Col-0 petal areas. Student's *t*-test showed no significant differences between the transformants and the parental *da1-ko1 dar1-1* line. (*I*) Transgenic *Arabidopsis* plants expressing a GFP-DA1 fusion protein under the control of the 35S promoter were used to detect DA1 ubiquitylation in vivo. GFP ran as a dimer on the gel due to high protein concentrations. Protein extract input levels are shown using anti-tubulin antibody.

The interactions of DA1 with BB and DA2 suggested that DA1 might be a substrate of these RING E3 ligases, so we conducted in vitro ubiquitylation reactions using BB, DA2, and BBR E3 ligases. Figure 2A shows that BB ubiquitylated DA1 in an E1- and E2-dependent reaction, as did DA2 (Fig. 2B), while BBR did not (Fig. 2C). Supplemental Figure S1 shows that DA2 also ubiquitylated DAR1 and DAR2 but not DAR3. The extent of DA1 ubiquitylation suggested that DA2 was more efficient at ubiquitylation than BB, and the sizes of ubiquitylated DA1 indicated that between four and seven ubiquitin molecules may be conjugated to DA1. Mass spectrometric analyses of ubiquitylated DA1 prepared in vitro were used to identify peptides containing the characteristic diglycine ubiquitylation signature of a lysine residue (KGG). Analysis of DA1 ubiquitylated by DA2 or BB identified seven ubiquitylated lysine residues in DA1, with four lysines in the C-terminal domain of DA1 (K381, K391, K475, and K591) consistently conjugated with ubiquitin (Supplemental Fig. S2). This number of ubiquitylation sites concurred with the patterns of ubiquitylation observed in Figure 2, A and B, suggesting that DA1 molecules are multiply ubiquitylated (Haglund et al. 2003; Komander and Rape 2012). Mutation of the consistently ubiquitylated lysines to arginine in DA1 [termed DA1(4K-4R)] did not reduce ubiquitylation by DA2 in vitro (Fig. 2D), and mass spectrometric analyses showed ectopic ubiquitylation of other lysines across DA1 (Supplemental Fig. S2B). Therefore, the DA1 ubiquitylation mechanism has a preference, but not specificity, for certain lysines. These patterns of ubiquitylation are shown in Figure 2D.

DA1 and four other family members have multiple ubiquitin interaction motifs (UIMs) that interact with ubiquitin (Li et al. 2008; Peng et al. 2015). UIMs are part of a larger class of ubiquitin-binding domains (UBDs) formed from a single α helix that is often found in multiple arrays (Hicke et al. 2005; Husnjak and Dikic 2012). Tandem UIMs have been shown to bind K63-linked ubiquitin chains in the mammalian DNA repair protein RAP80 (Sato et al. 2009). To assess their function in DA1, the N-terminal region of DA1 containing mutated UIM1 and UIM2 was fused to GST and expressed in *Escherichia coli*, and conserved Ala and Ser residues, predicted to be in the α-helical domain of the UIMs (Supplemental Fig. S3; Kim et al. 2007), were mutated to Gly in both UIMs of GST-UIM1+2 and in DA1. GST-UIM1+2 bound ubiquitin, and mutation of UIM1 alone did not reduce binding of ubiquitin, while mutation of UIM2 abolished ubiquitin binding, confirming that the GST-UIM1+2 protein bound ubiquitin via its UIM motifs (Supplemental Fig. S3). Figure 2E shows that UIM1+2 conferred BB- and DA2-dependent ubiquitylation on GST in vitro, with DA2 again facilitating higher levels of ubiquitylation. Figure 2F shows that mutation of both UIM1 and UIM2 in GST-UIM1+2 strongly reduced in vitro ubiquitylation of GST-UIM1+2 by BB and DA2. The UIM1 and UIM2 mutations in DA1 also reduced its ubiquitylation in vitro (Fig. 2G), and DA1 with mutated UIMs did not complement the large petal size in the double mutant *da1-ko dar1-1* (Fig. 2H). To detect ubiquitylation in vivo, DA1 was expressed from the constitutive 35S promoter as an N-terminal GFP fusion protein and purified from seedling tissues using a GFP trap. Characteristic patterns of DA1 ubiquitylation were detected on purified GFP-DA1 (Fig. 2I, right panel). Therefore, DA1 is ubiquitylated by the E3 ligases BB and DA2 in vitro by a UIM1- and UIM2-dependent mechanism, DA1 is ubiquitylated in vivo, and UIMs are required for DA1 function.

### DA1 cleaves BB and DA2 with a ubiquitin-dependent peptidase activity

**Figure 3.**
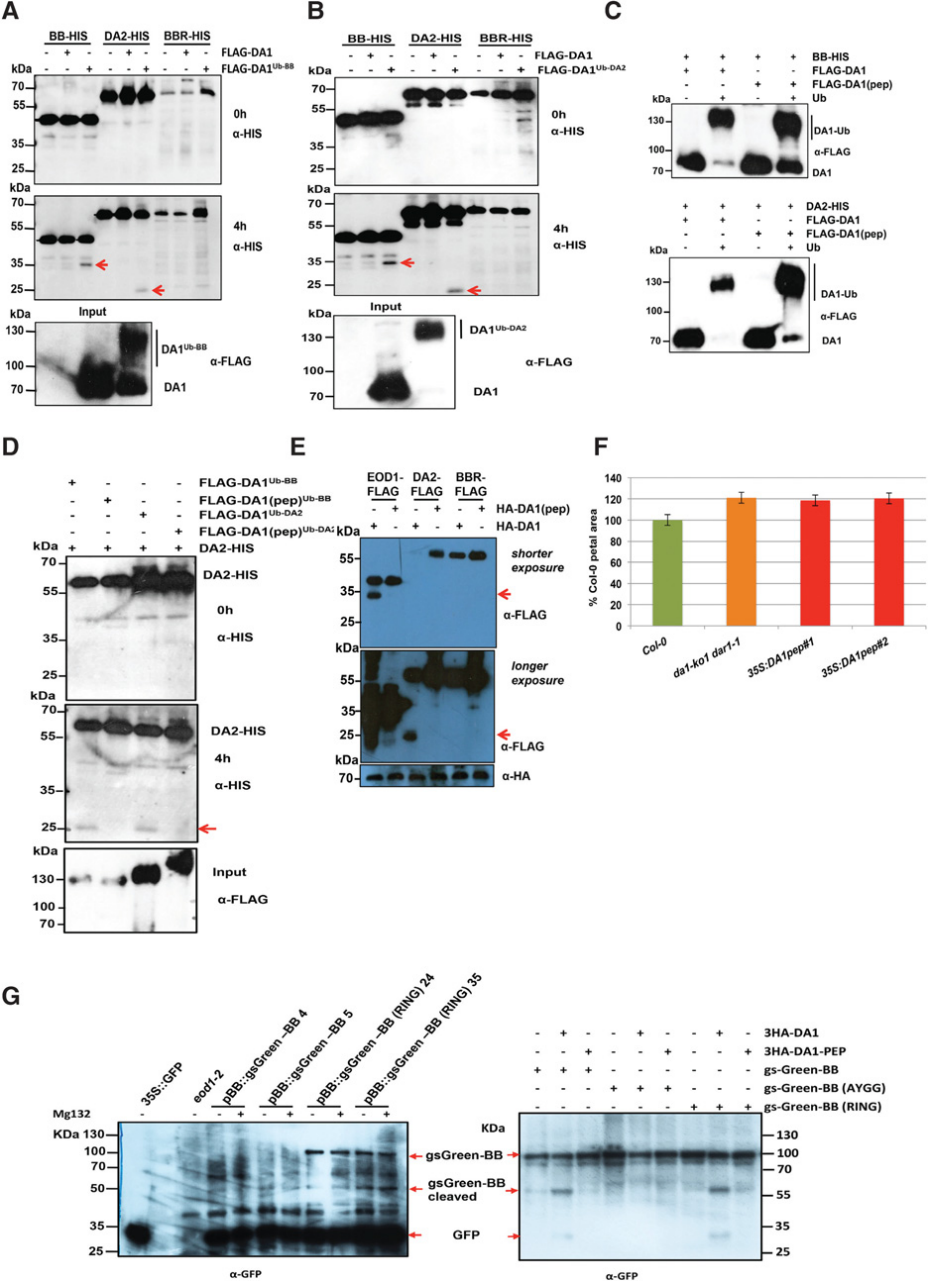
DA1 is an endopeptidase activated by multiple ubiquitylations and cleaves the E3 ligases BB and DA2 that ubiquitylate it. (*A*,*B*) Time course of an in vitro reaction of Flag-DA1 or Flag-DA1 ubiquitylated by BB (*A*) or DA2 (*B*) with BB-HIS, DA2-HIS, and BBR-HIS. The *bottom* panels show loading of Flag-DA1 and Flag-DA1^Ub-BB^. After 4 h, cleavage products (shown by red arrows) of BB and DA2 had been produced by ubiquitylated Flag-DA1 but not Flag-DA1. BBR was not cleaved under these conditions. (*C*) An in vitro ubiquitylation reaction of DA1 and DA1(pep) using BB-HIS (*top* panel) and DA2-HIS (*bottom* panel) as E3 ligases. Ubiquitin-dependent multiple monoubiquitylations of Flag-DA1 and Flag-DA1(pep) by both BB and DA2 were detected. (*D*) A time course of an in vitro cleavage reaction using DA2-HIS as a substrate (*left* panels) and Flag-DA1 or Flag-DA1(pep) ubiquitylated by either BB or DA2 (loading shown in the *bottom* panel). The red arrow in the *bottom left* panel indicates the DA2 cleavage product at 4 h that was produced only by Flag-DA1^Ub^ and not Flag-DA1(pep)^Ub^. (*E*) *Arabidopsis da1-ko1 dar1-1* mesophyll protoplasts were cotransfected with plasmids expressing BB-Flag, DA2-Flag, BBR-Flag, HA-DA1, and HA-DA1(pep). The same-sized cleavage products (red arrows) from BB-Flag and DA2-Flag were detected as seen in *A* and *B* above. (*Middle* panel) Longer exposure of the *top* immunoblot showed cleaved DA2. The *bottom* panel shows loading of HA-DA1 and HA-DA1(pep). (*F*) DA1(pep) is not functional in vivo, as it does not complement the large petal phenotype of the *da1-ko1 dar1-1* double mutant. Transformants expressing *35S::DA1(pep)* were scored for petal size and compared with wild-type Col-0 and *da1-ko1 dar1-1*. The values given are means (*n* = 150) ± SE, expressed as percentage of wild-type Col-0 petal areas. Student's *t*-test showed no significant differences between the transformants and the parental *da1-kodar1-1* line. (*G*) Cleavage of gsGreen-BB is shown in planta in the *left* panel and in transiently expressed protoplasts in the *right* panel for comparison. Large-scale protein extracts from transgenic 8-d-old seedlings expressing *BB::gsGreen-BB* and *BB::gsGreen-*BB (RING) were purified on a GFP trap. Loading controls used levels of free GFP. The expected size cleavage products (arrows) were observed in plant extracts and protoplasts for comparison.

A time course of BB-HIS incubated with purified Flag-DA1 that had been ubiquitylated by BB or incubated with nonubiquitylated Flag-DA1 showed that, in the presence of ubiquitylated DA1, a HIS-tagged BB fragment of ∼35 kDa was produced after 4 h of incubation (Fig. 3A, arrows). When ubiquitylated Flag-DA1 was incubated with DA2-HIS, a 25-kDa HIS-tagged DA2 cleavage product was also detected after 4 h of incubation (Fig. 3A, arrows). Similar experiments using Flag-DA1 ubiquitylated by DA2 showed identical patterns of BB-HIS and DA2-HIS cleavage (Fig. 3B). BBR-HIS did not show a cleavage product in these conditions. Thus, DA1 ubiquitylated by either BB or DA2 generated cleavage products from both BB and DA2 in vitro.

Examination of the conserved C-terminal region of DA1 revealed an extended sequence motif, HEMMHX_15_EE (Supplemental Fig. S4), which is a zinc aminopeptidase active site found in clan MA endopeptidases (Rawlings et al. 2012). The HEMMH motif was mutated to AEMMA, removing the putative zinc-coordinating histidine residues, to form DA1(pep). Figure 3C shows that DA1(pep) and DA1 were ubiquitylated in vitro to an equal extent by both BB and DA2. In an in vitro time-course reaction, ubiquitylated DA1(pep) did not generate the 25-kDa HIS-tagged DA2 band seen after incubation with ubiquitylated DA1 (Fig. 3D). Coexpression of BB-Flag, DA2-Flag, or BBR-Flag with HA-DA1 or HA-DA1(pep) in *da1-ko1 dar1-1* mutant leaf protoplasts showed that HA-DA1, but not HA-DA1(pep), generated a similar-sized 35-kDa BB-Flag cleavage product (Fig. 3E, top panel, arrow) as seen in in vitro reactions (Fig. 3A,B). Longer exposure of the same Western blot (Fig. 3E, bottom panel) was required to identify the 25-kDa DA2-Flag cleavage product, which was not generated by coexpression with DA1(pep). Figure 3F shows that the mutation in *DA1* abolishing DA1 peptidase activity did not complement the *da1-ko1 dar1-1* large petal phenotype, establishing that DA1 peptidase activity is required for in vivo function. To detect DA1 peptidase activity in vivo, transgenic plants expressing *BB::gsGreen-BB* gene fusion and a RING domain mutant version that was predicted to be more stable in vivo due to reduced autopolyubiquitylation (Disch et al. 2006) were generated. Analysis of GFP trap-purified proteins (Fig. 3G, left panel) showed a cleavage product of the expected size generated from RING mutant gsGreen protein in two independent transformants. Full-length wild-type gsGreen-BB was not detected, although low levels of an expected cleavage product were identified. For comparison, the same constructs, together with a noncleavable form (AY-GG) (see Fig. 5B, below), were expressed using the 35S promoter in protoplasts with DA1 (Fig. 3G, right panel). This showed the predicted DA1-mediated BB cleavage product, which was not generated in the AY-GG version of BB.

**Figure 4.**
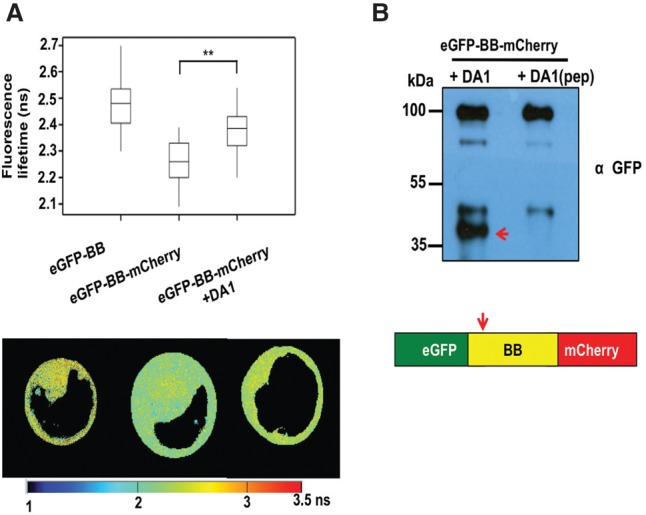
Detection of DA1-mediated cleavage of BB in vivo using FRET. (*A*) Root protoplasts of *da1-ko1 dar1-1* plants were transfected with the FRET construct eGFP-BB or a control eGFP-BB-mCherry construct together with DA1 to detect DA1-mediated cleavage of BB. Transfected 2protoplasts were imaged using multiphoton microscopy, and fluorescence half-times of protoplasts (*n* = 13) were captured. The heat map shows fluorescent lifetime values, and typical protoplasts are shown to illustrate fluorescent half-lives imaged over the cell. The box plots show significantly increased fluorescence lifetime after DA1 transfection. (**) *P* ≤ 0.001, Student's *t*-test. (*B*) Cleavage of eGFP-BB-mCherry by DA1 in the imaged protoplasts shown in *A*. The arrow shows the major cleavage product of ∼40 kDa expected from DA1 cleavage near the N terminus of BB.

A Förster resonance energy transfer (FRET) DA1 peptidase sensor was constructed using eGFP donor and mCherry acceptor pairs (van der Krogt et al. 2008) connected by BB to provide another measure of DA1 peptidase activity in vivo. Cleavage of the fluorophore pair by DA1 would increase the fluorescence lifetime toward that of eGFP-BB compared with that of the intact sensor protein by impairing energy transfer between the fluorophores. The peptidase sensor and a control donor sensor were transfected into *da1-ko1 dar1-1* root protoplasts, and fluorescence lifetime imaging (FLIM) was performed. Figure 4A shows that the fluorescence lifetime (τ) of the GFP-BB donor control was ∼2.48 nsec, while that of an intact donor–acceptor pair was ∼2.25 nsec, demonstrating efficient FRET. When cotransfected with DA1, the fluorescent lifetime of the donor–acceptor pair increased to ∼2.38 nsec. Lifetime imaging of typical transfected protoplasts showed a generalized cellular localization of DA1-mediated cleavage. Figure 4B shows that the eGFP-BB-mCherry donor–acceptor pair was cleaved by DA1 peptidase at the expected site in transfected root protoplasts. Therefore, DA1 has a latent peptidase activity that is activated by multiple ubiquitylation mediated by its UIM1+2 domain and the RING E3 ligases BB and DA2, and activated DA1 peptidase then specifically cleaves these two E3 ligases.

### Identification of a DA1 peptidase cleavage site in BB

**Figure 5.**
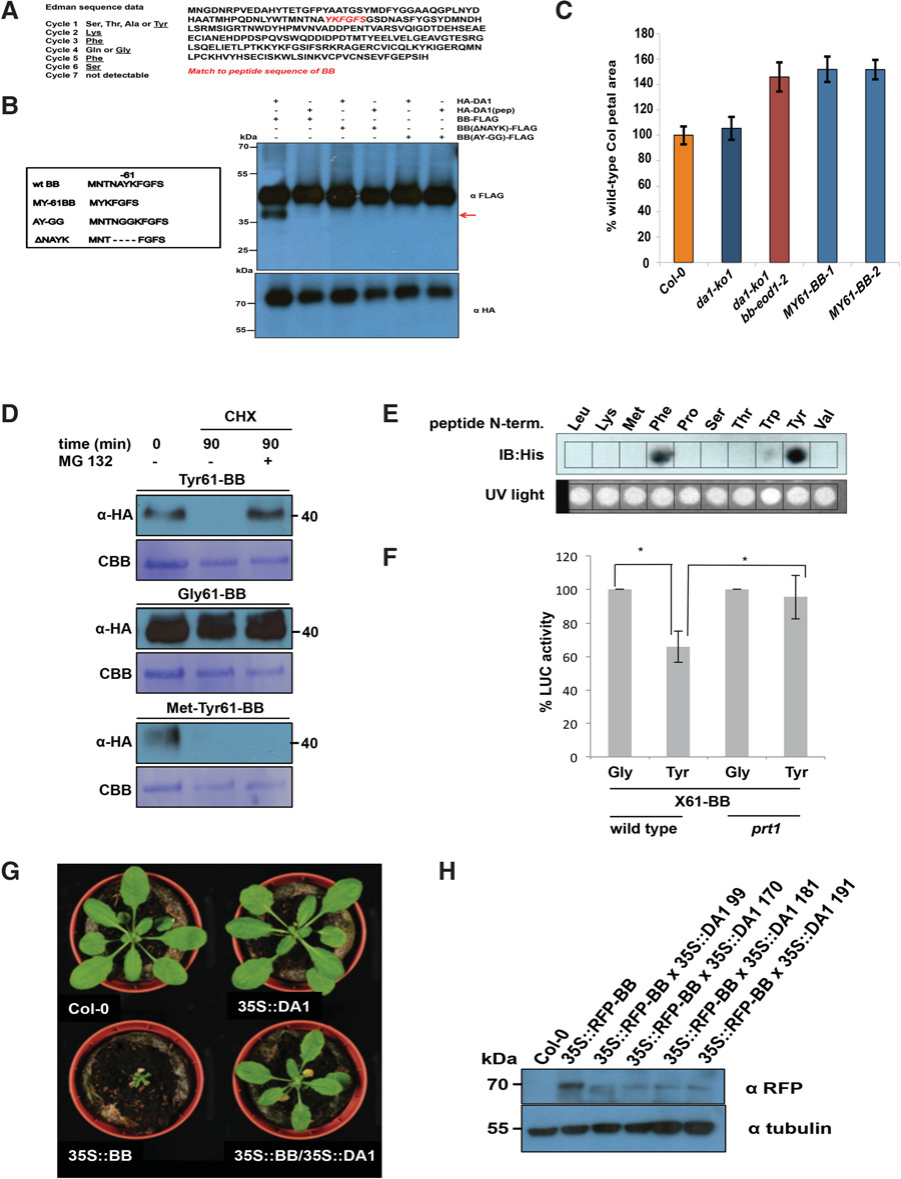
Identification of the DA1 cleavage site in BB and destabilization and functional inactivation of cleaved BB in vivo by the N-end rule. (*A*) Neo-N-terminal sequences of purified cleaved BB-HIS (*left*) matched the complete BB protein sequence (*right*, shown in red). Data from six Edman sequencing cycles are in Supplemental Figure S5. (*B*) The predicted DA1 cleavage site in BB was mutated by changing the AY amino acids flanking the site to GG (AY-GG) and deleting two amino acids from both sides of the predicted cleavage site (ΔNAYK). BB-Flag, BB (ΔNAYK)-Flag, and BB (AY-GG)-Flag were expressed in *da1-ko1 dar1-1 Arabidopsis* mesophyll protoplasts under the control of the 35S promoter. HA-DA1 did not cleave BB containing mutations in the predicted cleavage site. The *bottom* panel shows HA-DA1 and HA-DA1(pep) loading. (*C*) A DA1-cleaved version of BB does not function in planta. A cleaved version of BB, termed MY61-BB, was expressed in *da1-ko1 bb-eod1-2* plants under control of the 35S promoter. The graph compares petal areas in wild-type Col-0, *da1-ko1*, *da1-ko1 bb-eod1-2*, and two independent transgenic lines. Values give are means (*n* = 50) ± SE, expressed as percentage of wild-type Col-0 petal areas. Student's *t*-test showed no significant differences between the transformants and the parental *da1-ko1 bb-eod1-2* line. (*D*) In vitro degradation of BB is dependent on N termini. Ubiquitin fusion constructs were expressed in a reticulocyte lysate cell-free system. Samples were incubated for 30 min with or without MG132. Next, cycloheximide (CHX) was added to inhibit translation, and samples were taken 0 and 90 min after CHX addition. Samples were electrophoresed on SDS-PAGE and immunoblotted using HA antibodies to detect BB protein levels. Loading controls were the CBB-stained membrane. (*E*) PRT1 binding to synthetic peptides mimicking the neo-N-terminal of 61BB. SPOT assay of a peptide array of synthetic 17-mer peptides incubated with recombinant His8-MBP-tagged PRT1. Peptides were derived from an N-recognin test substrate, and the first amino acid comprised Leu, Lys, Met, Phe, Pro, Ser, The, Trp, Tyr, and Val. His-PRT1 was detected by immunoblotting. Equal peptide loading on the membrane was monitored by UV light prior to PRT1 protein binding. (*F*) Constructs expressing ubiquitin fusions of 61BB-Luciferase constructs with glycine or tyrosine neo-N termini (Ub-Gly-61-BB-HA-LUC and Ub-Tyr-61-BB-HA-LUC) were transfected into wild-type or *prt1* mutant protoplasts. Transfection efficiency was measured using a pUBC::GUS control. Luciferase activities were normalized to GUS activity, and the luciferase activity of Gly-61-BB-HA-LUC was taken as 100%. The significance of differences was calculated from three independent transformation experiments using Student's *t*-tests (two sites, uncoupled). (*) *P*-value ≤ 0.05. (*G*) Overexpression of BB under the control of the 35S promoter leads to strongly reduced growth, and, when crossed with a line overexpressing DA1, this growth inhibition was reversed. This demonstrated that DA1 can reduce the growth inhibitory effect of high levels of BB. (*H*) Crossing a line overexpressing DA1 into a line expressing 35S::RFP-BB reduced RFP-BB levels. Homozygous progeny of four independent crosses (99, 170, 181, and 191) are shown.

To define the potential functions of DA1-mediated cleavage, the DA1 cleavage site in BB was identified using Edman sequencing of purified cleaved BB-HIS. Supplemental Figure S5 shows neo-N-terminal amino acid sequences that had a unique match to six amino acids in BB (Fig. 5A). This indicated a potential DA1 cleavage site within BB between A_60_ and Y_61_, consistent with the sizes of BB and its ∼35-kDa cleaved form (Fig. 3A). Two mutant forms of BB were made to assess this potential DA1 cleavage site: a four-amino-acid deletion surrounding the site (ΔNAYK) and AY changed to GG (AY-GG) (Fig. 5B). These proteins were coexpressed in *Arabidopsis da1-ko1 dar1-1* mesophyll protoplasts as C-terminal Flag fusion proteins with HA-DA1 and HA-DA1(pep). Figure 5B shows that the mutant BB-Flag proteins were not cleaved by DA1, establishing that DA1 peptidase activity cleaved BB between A_60_ and Y_61_. A cleaved form of BB, called MY61-BB, was also made with an initiator Met followed by Y_61_ (Fig. 5B). MY61-BB was expressed using the 35S promoter in *da1-ko1 bb-eod1-2* mutant *Arabidopsis*. Its lack of complementation of *bb-eod1-2* (Fig. 5C) showed that DA1 peptidase-mediated cleavage reduced BB activity.

### BB stability is dependent on it N terminus and N-end rule function

DA1 cleavage products of DA2 were unstable, indicating that one function of DA1-mediated cleavage may be to destabilize proteins (Fig. 3E). This was also observed for BB in cell-free degradation assays, in which MY61-BB was unstable compared with wild-type BB (Supplemental Fig. S6). To test the role of the neo-N terminus of BB on protein stability, 61BB proteins with different N termini (Y, G, and MY) were expressed using the ubiquitin fusion technique (UFT) (Bachmair et al. 1986). HA-tagged constructs were translationally coexpressed in a cell-free rabbit reticulocyte system with or without MG132 proteasome inhibitor, and translation was stopped by the addition of cycloheximide. Y61-BB was highly unstable, whereas G61-BB was stable (Fig. 5D). Interestingly, the artificial MY61-BB was also highly unstable in a proteasome-independent mechanism. The neo-N-terminal sequence of DA1-cleaved BB starts with YK, a potentially destabilizing sequence of a type II N-end rule degron (Varshavsky 2011). The N-end rule E3 ligase PRT1 mediates the stability of model N-end rule substrates with such aromatic N-terminal residues (Potuschak et al. 1998). To assess the potential role of PRT1 in N-end rule-mediated degradation of BB, we tested the binding of PRT1 to 17-mer peptides representing variants of the neo-N termini of BB on a backbone sequence of an N-end rule test substrate in SPOT (synthetic peptide arrays on membrane support technique) assays. Purified recombinant HIS-MBP-PRT1 protein was incubated with the SPOT array, and binding was visualized by Western blotting. Recombinant PRT1 had a preference for binding to the large aromatic acids tyrosine and phenylalanine, consistent with previously suggested specificity (Fig. 5E; Potuschak et al. 1998; Stary 2003; Faden et al. 2016). To assess whether PRT1 had a role in DA1-mediated BB degradation, BB was expressed with an N-terminal ubiquitin fusion and a C-terminal luciferase fusion to reveal neo-N termini in Col-0 or *prt1* mutant mesophyll protoplasts. BB-LUC activity was reduced in wild-type protoplasts with a neo-N-terminal tyrosine, which was not seen in *prt1* mutant protoplasts (Fig. 5F). Neo-N-terminal glycine BB-LUC levels were not altered in either Col-0 or *prt1* mutant protoplasts. This indicated a strong dependence of Tyr-61BB stability on PRT1 activity. In planta evidence supporting the role of DA1 in reducing the growth inhibitory role of BB via N-end rule-mediated degradation was shown by the suppression of growth reduction in a transgenic 35S::*RFP-BB* overexpression line by overexpression of DA1 (Fig. 5G). Western blots (Fig. 5H) confirmed that 35S::DA1 reduced levels of RFP-BB.

### Functional analyses of DA1

**Figure 6.**
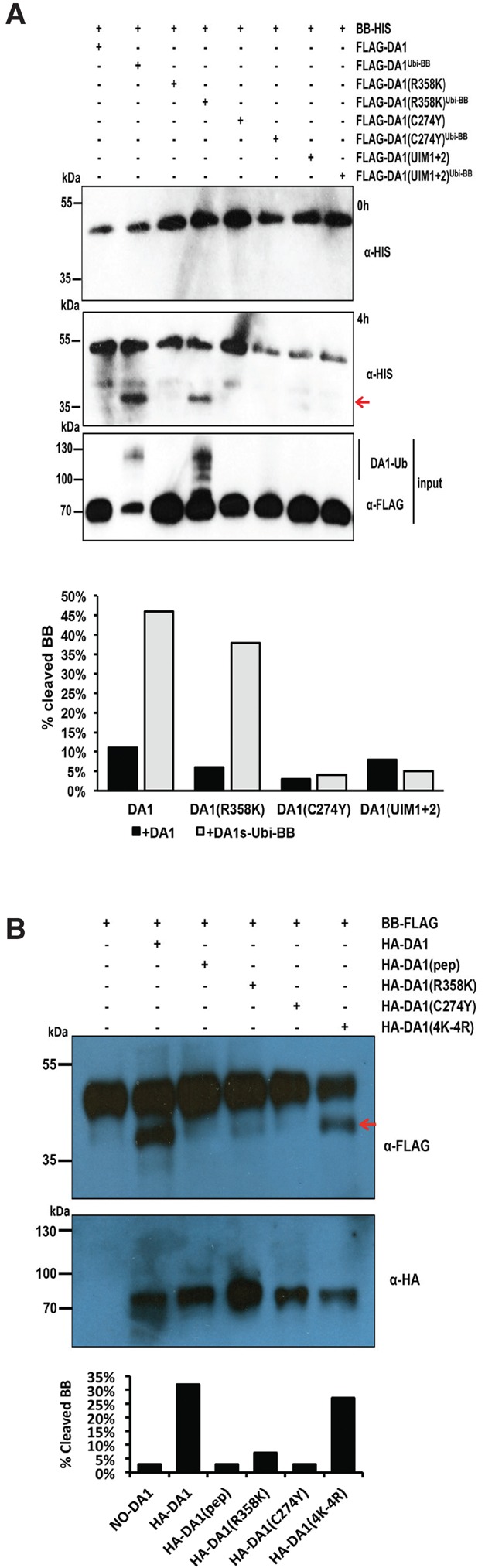
Functional analyses of DA1 activity in vitro and in vivo. (*A*) In vitro DA1 cleavage assays using BB-HIS as a substrate for ubiquitylated Flag-DA1 and mutant versions. The *top* panel shows the reaction at 0 h, and the *middle* panel shows the reactions at 4 h. The red arrow indicates cleaved BB-HIS. The loading and ubiquitylation status of input HA-DA1 and mutant versions is shown. Quantitative scans showed that BB-HIS was cleaved to a greater extent by wild-type DA1-HIS than by HA-DA1(R358K). HA-DA1(C274Y) and HA-DA1(UIM1+2) did not cleave BB-HIS. (*B*) In vivo DA1 cleavage reactions using BB-Flag and HA-tagged DA1 and mutant versions. The BB-Flag cleavage product is indicated by an arrow. Quantitative scans showed reduced levels of BB cleavage by HA-DA1(R358K), and HA-DA1(C274Y) did not cleave BB-Flag. HA-DA1(4K-4R) had normal levels of peptidase activity. The loading of HA-DA and its mutant versions is shown in the *bottom* immunoblot.

We showed previously that the *da1-1* allele of *DA1* has a negative interfering phenotype with respect to the closely related family member *DAR1* (Li et al. 2008). The peptidase activity of the protein encoded by the *da1-1* allele, called DA1(R358K), which has an arginine to a lysine residue altered in a highly conserved C-terminal region, (Supplemental Fig. S4) was assessed. This mutation did not influence ubiquitylation of Flag-DA1(R358K) (Fig. 6A) or create a site for ectopic ubiquitylation of Flag-DA1(R358K), as determined by mass spectrometric analysis (Supplemental Fig. S2C). The peptidase activity of ubiquitylated Flag-DA1(R358K) was qualitatively assessed in vitro and in vivo [using HA-DA1(R358K)] by comparison with wild-type DA1 peptidase activity (Fig. 6A,B). Both assays showed that DA1(R358K) had lower peptidase activity compared with DA1, suggesting that regions of the conserved C-terminal region are required for peptidase activity and that the *da1-1* phenotype may be due to reduced peptidase activity. Figure 6B also shows that DA1(4K-4R), which is ubiquitylated (Fig. 2E), had peptidase activity toward BB. This suggested that precise patterns of ubiquitylation are not required for activating DA1 latent peptidase activity.

*DAR4*, another *DA1* family member (Li et al. 2008), encodes a protein with an N-terminal TIR-NB-LRR and has a gain-of-function *chs3-2d* allele in the conserved C-terminal region (Supplemental Fig. S3) that activated constitutive defense responses (Xu et al. 2015). Alignments revealed high similarity to predicted protein sequences from the photosynthetic bacteria *Roseiflexus* sp (Supplemental Fig. S7; Burroughs et al. 2011) that included four pairs of CxxC/H motifs with the potential to bind zinc similar to those in canonical LIM domains (Kadrmas and Beckerle 2004). The *chs3-2d* mutation changes a cysteine to a tyrosine in the third pair of conserved CxxC/H motifs (Supplemental Figs. S3, S6), suggesting that it may alter a possible LIM-like structure. This mutation was introduced into DA1 to create DA1(C274Y), and its activities were assessed. Figure 6A shows that DA1(C274Y) was not ubiquitylated by BB and had no peptidase activity toward BB in vitro and in vivo (Fig. 6B). This implicated the putative LIM-like domain in DA1 in UIM-mediated ubiquitylation and activation of DA1 peptidase activity.

### DA1 peptidase activity cleaves TCP15, TCP22, and UBP15

**Figure 7.**
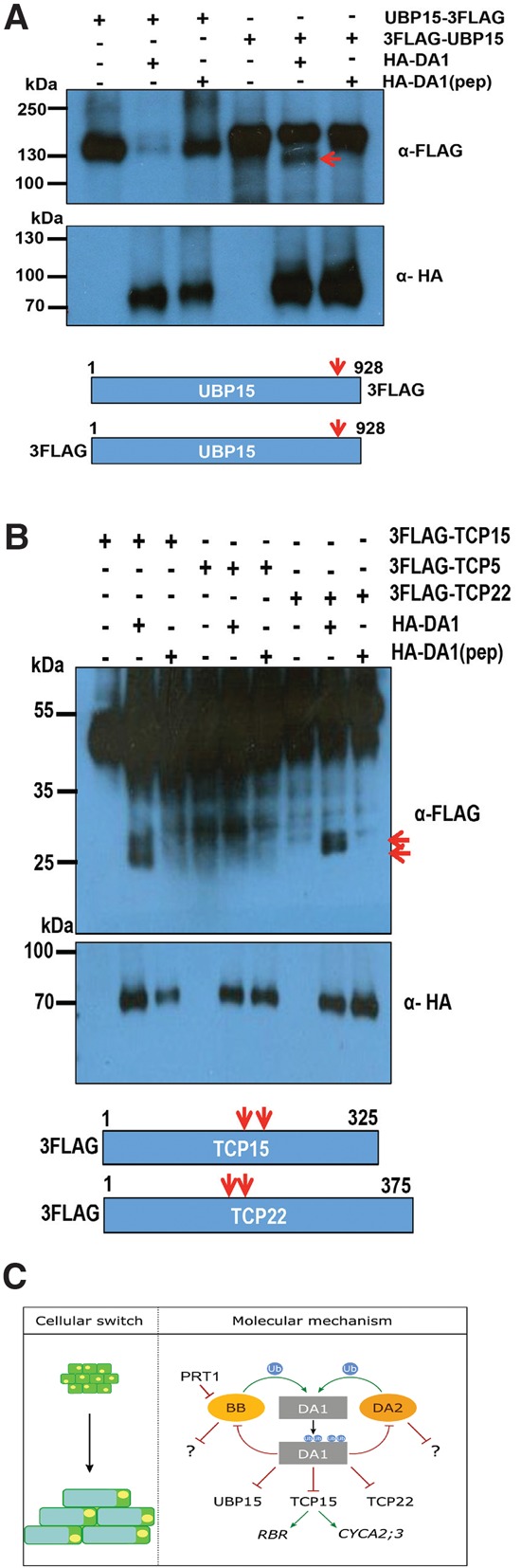
DA1 cleaves UBP15, TCP15, and TCP22 in vivo. (*A*,*B*) In vivo cleavage reactions of UBP15-3Flag and 3Flag-UBP15 (*A*) as well as 3-Flag-TCP15, 3-Flag-TCP22, and 3-Flag TCP5 (a noncleaved control) (*B*) using HA-DA1 and HA-DA1(pep). Constructs expressed from the 35S promoter were cotransfected into *da1-ko1 dar1-1* mesophyll protoplasts. UBP15, TCP14, TCP15, and TCP22 cleavage products are shown in the *top* immunoblots. The *bottom* immunoblots show HA-DA1 and HA-DA1(pep) protein levels. The approximate locations of DA1 cleavage sites (arrows) are shown in UBP15, TCP15, and TCP22. (*C*) A model of the proposed transient mechanism of DA1 peptidase activation and the consequences of DA1-mediated cleavage of growth regulators during organ growth. PRT1 activity is shown as degrading BB.

The increased levels of UBP15 (Du et al. 2014), TCP14, and TCP15 proteins (Peng et al. 2015) observed in the *da1-1* mutant suggested that DA1 activity may reduce the stability of these proteins by peptidase-mediated cleavage. Figure 7A shows that DA1 peptidase cleaved UBP15 close to its C terminus when transiently expressed together in *da1-ko1 dar1-1* protoplasts. The reduced signal in the Western blot with the C-terminal Flag fusion was due to the short Flag-tagged protein running off the gel. TCP15 and the closely related TCP22 proteins were also cleaved by DA1 in protoplasts (Fig. 7B), but we could not consistently detect TCP14 cleavage by DA1 or DAR1. These data show that DA1 peptidase activity can cleave UBP15, which promotes cell proliferation, and TCP15, which inhibits endoreduplication (Du et al. 2014; Peng et al. 2015).

## Discussion

### DA1 is a latent peptidase activated by multiple ubiquitylation

The addition of ubiquitin molecules to substrate proteins is a common post-translational modification with many regulatory roles (Haglund and Stenmark 2006; Hoeller and Dikic 2010). We showed that DA1 was ubiquitylated at four lysine residues (Fig. 2A–D; Supplemental Fig. S2) by the E3 ligases BB and DA2, which genetically (Fig. 1A–D) and physically (Fig. 1D,E) interact with DA1. Similar levels of DA1 ubiquitylation were observed in vivo (Fig. 2I). Loss-of-function mutants of *BB* and *DA2* synergistically increased the large organ phenotypes of a *DA1* loss-of-function mutant (Fig. 1A–D; Li et al. 2008; Xia et al. 2013). Therefore, ubiquitylation mediated by these two E3 ligases may increase the growth-limiting activity of DA1. Quantitative growth measurements of leaves (Fig. 1E) showed that, individually, the *da1-1* mutant and *bb-eod1-2* mutants had distinctive effects on leaf growth; *da1-1* had a delayed time of maximum growth rate, while *bb-eod1-2* showed a slightly accelerated time of maximum growth compared with Col-0. In combination, the mutants showed an even greater maximum growth rate and delayed time of maximum growth rate, increasing final organ size. BB and DA2 act independently to influence final organ size (Xia et al. 2013), but they both also interact with DA1 (Fig. 1F,G; Li et al. 2008; Xia et al. 2013), indicating that BB and DA2 may ubiquitylate different substrates, perhaps for proteasomal-mediated degradation. Quantitatively, loss-of-function mutations in *BB* and *DA2* show that their independent effects on growth are less than that of *da1-1* (Fig. 1A–D).

Members of the DA1 family have a canonical MA clan zinc metallopeptidase active site domain in their conserved C-terminal regions (Supplemental Fig. S4; Tholander et al. 2010; Rawlings et al. 2012) that was required for limiting growth (Fig. 3F). BB- and DA2-mediated ubiquitylation activated DA1 peptidase activity in vitro and in transiently expressed protoplasts (Fig. 3A,E), establishing a biochemical foundation for their joint activities in growth control. Zinc metallopeptidases are maintained in an inactive form by a “cysteine switch” (Van Wart and Birkedal-Hansen 1990) that coordinates a cysteine residue with the zinc atom at the active site to block it. Conformational changes release this and activate the peptidase.

Ubiquitylation of DA1 has the potential to trigger a conformational change that may release inhibition of peptidase activity. Hoeller et al. (2006) showed that UBD- and UIM-mediated monoubiquitylation of endocytotic proteins, including epsin, led to a conformational change mediated by intramolecular interactions between UBDs/UIMs and *cis*-ubiquitin, which regulated endocytosis. The binding of ubiquitin to DA1 UIMs was required for DA1 function in vivo (Fig. 2H), and the UIMs conferred patterns of ubiquitylation on the heterologous protein GST similar to that seen for DA1 (Fig. 2G [for DA1], E,F [for GST-UIM1+2]). Related observations were seen in the monoubiquitylation of epsin (Oldham et al. 2002) through coupled monoubiquitylation (Woelk et al. 2006), where UIMs recruit the UIM-containing protein to the ubiquitylation machinery by direct interaction with ubiquitin coupled to ubiquitin donor proteins (Haglund and Stenmark 2006). Mutation of Cys274 in the C-terminal zinc finger loop of the LIM-like domain of DA1 abrogated both ubiquitylation and peptidase activity (Fig. 6A,B), suggesting a functional role for this ancient conserved LIM-like domain (Supplemental Fig. S7; Burroughs et al. 2011) in peptidase activation. Analyses of conformational changes caused by DA1 ubiquitylation and their influence on peptidase activity are required to establish this potential mechanism.

### DA1 cleavage destabilizes its activating E3 ligases (BB and DA2), and cleavage of BB leads to targeting by the N-recognin PRT1

The RING E3 ligases BB and DA2 activate DA1 peptidase by ubiquitylation and are also cleaved by DA1 peptidase (Figs. 3A,B,E,G, 4). Once cleaved, DA2 appeared to be destabilized in transiently expressed protoplasts (Fig. 3E). Identification of the DA1 cleavage site in BB (Fig. 5A,B) revealed Y61-BB at the neo-N terminus of cleaved BB. This neo-N terminus conferred proteasome-mediated degradation in a cell-free system (Fig. 5D). This degradation depended on recognition of the neo-N terminus by the *Arabidopsis* E3 ligase PRT1 (Fig. 5E,F; Potuschak et al. 1998; Stary 2003), an N-recognin catalyzing N-end rule-mediated degradation (Varshavsky 2011) with a suggested preference for aromatic amino acid N termini. Interestingly, the neo-N-terminal MY61-BB, which was used to express a cleaved version of BB in planta, conferred strong proteasome-independent instability (Fig. 5D) in a mechanism that is not yet clear. The lack of MY61-BB function in vivo (Fig. 5C) supported the observation that DA1-mediated cleavage of BB leads to its loss of function in vivo. Overexpression of *BB* strongly reduced growth, as expected from its inhibitory role in growth (Disch et al. 2006). The reversal of this inhibition by overexpression of *DA1*, which reversed growth inhibition (Fig. 5G) and reduced RFP-BB levels (Fig. 5H), is consistent with a mechanism involving DA1-mediated reduction of BB activity via peptidase-mediated cleavage and subsequent degradation by the N-end rule pathway. Such an activation–destruction mechanism mediated by BB, DA2, and DA1 may provide a way of tightly controlling peptidase activity. The physiological role of these mechanisms, which often involve ubiquitylation and proteolytic degradation, is to drive unidirectional cellular processes; for example, in cell cycle progression (Reed 2003). The factors that trigger DA1 ubiquitylation by BB and coordinate the activities of BB and DA2 remain unknown.

### DA1 peptidase activity also cleaves diverse growth regulators

We showed previously that *TCP14* and *TCP15* function downstream from DA1 and other family members in controlling organ size in *Arabidopsis*, and reduced function of DA1 family members led to increased TCP14 and TCP15 protein levels (Peng et al. 2015). Similarly, levels of UBP15 protein, which promotes cell proliferation (Liu et al. 2008) and also functions downstream from DA1, were increased in the *da1-1* reduced-function mutant (Du et al. 2014). We showed that TCP15 and the related TCP22 as well as UBP15 were cleaved by DA1 peptidase activity (Fig. 7A,B) but could not reliably detect TCP14 cleavage by DA1 or DAR1. DA1-mediated cleavage of TCP15 and UBP15 is a plausible mechanism that accounts for these observed reduced protein levels, similar to DA1-mediated inactivation and destabilization of BB by peptidase cleavage. Taken together, these observations suggest a mechanism (Fig. 7C) in which DA1 peptidase, activated transiently by BB or DA2, coordinates a “one-way” cessation of cell proliferation and the initiation of endoreduplication through the cleavage and potential inactivation of proteins that promote cell proliferation and inhibit endoreduplication.

## Materials and methods

### Plant materials, growth conditions, and organ size measurements

*A. thaliana* Col-0 was the wild-type plant used. Plants were grown in growth rooms at 20°C with 16-h day/8-h dark cycles using either soil or MS medium supplemented with 0.5% glucose. Petal and seed areas were imaged by high-resolution scanning (3600 dpi; Hewlett Packard Scanjet 4370) and analyzed using ImageJ software (http://rsbweb.nih.gov/ij).

### In vitro DA1-mediated cleavage assays

Flag-DA1 was ubiquitylated in vitro using either DA2-HIS or BB-HIS as E3 ligases, purified using Flag magnetic beads, and quantified, and 100 ng was added to 100 ng of BB-HIS, DA2-HIS, or BBR-HIS in a 30-µL reaction in 50 mM Tris HCl (pH 7.4) and 5 mM MgCl_2_. Reactions were carried out for 4 h at 30°C and terminated by the addition of SDS sample buffer.

### Mass spectrometry analysis

DA1 ubiquitylation patterns were determined from trypsinized proteins purified on SDS-PAGE gels. For liquid chromatography-tandem mass spectrometry analysis, peptides were applied to an LTQ-Orbitrab (Thermo-Fischer) using a nanoAcquity ultraperformance liquid chromatography system (Waters Ltd.). Further details are in the Supplemental Material.

## Supplementary Material

Supplemental Material

## References

[DONGGAD292235C1] Andriankaja M, Dhondt S, De Bodt S, Vanhaeren H, Coppens F, De Milde L, Mühlenbock P, Skirycz A, Gonzalez N, Beemster GTS, 2012 Exit from proliferation during leaf development in *Arabidopsis thaliana*: a not-so-gradual process. Dev Cell 22: 64–78. 22227310 10.1016/j.devcel.2011.11.011

[DONGGAD292235C2] Bachmair A, Finley D, Varshavsky A. 1986 In vivo half-life of a protein is a function of its amino-terminal residue. Science 234: 179–186. 3018930 10.1126/science.3018930

[DONGGAD292235C3] Barry ER, Camargo FD. 2013 The Hippo superhighway: signaling crossroads converging on the Hippo/Yap pathway in stem cells and development. Curr Opin Cell Biol 25: 247–253. 23312716 10.1016/j.ceb.2012.12.006

[DONGGAD292235C4] Breuer C, Ishida T, Sugimoto K. 2010 Developmental control of endocycles and cell growth in plants. Curr Opin Plant Biol 13: 654–660. 21094078 10.1016/j.pbi.2010.10.006

[DONGGAD292235C5] Breuninger H, Lenhard M. 2012 Expression of the central growth regulator BIG BROTHER is regulated by multiple *cis*-elements. BMC Plant Biol 12: 41. 22433627 10.1186/1471-2229-12-41 PMC3362746

[DONGGAD292235C6] Burroughs AM, Iyer LM, Aravind L. 2011 Functional diversification of the RING finger and other binuclear treble clef domains in prokaryotes and the early evolution of the ubiquitin system. Mol Biosyst 7: 2261. 21547297 10.1039/c1mb05061c PMC5938088

[DONGGAD292235C7] De Veylder L, Larkin JC, Schnittger A. 2011 Molecular control and function of endoreplication in development and physiology. Trends Plant Sci 16: 624–634. 21889902 10.1016/j.tplants.2011.07.001

[DONGGAD292235C8] Disch S, Anastasiou E, Sharma VK, Laux T, Fletcher JC, Lenhard M. 2006 The E3 ubiquitin ligase BIG BROTHER controls *Arabidopsis* organ size in a dosage-dependent manner. Curr Biol 16: 272–279. 16461280 10.1016/j.cub.2005.12.026

[DONGGAD292235C9] Du L, Li N, Chen L, Xu Y, Li Y, Zhang Y, Li C, Li Y. 2014 The ubiquitin receptor DA1 regulates seed and organ size by modulating the stability of the ubiquitin-specific protease UBP15/SOD2 in *Arabidopsis*. Plant Cell 26: 665–677. 24585836 10.1105/tpc.114.122663 PMC3967032

[DONGGAD292235C10] Efroni I, Blum E, Goldshmidt A, Eshed Y. 2008 A protracted and dynamic maturation schedule underlies *Arabidopsis* leaf development. Plant Cell 20: 2293–2306. 18805992 10.1105/tpc.107.057521 PMC2570723

[DONGGAD292235C11] Faden F, Ramezani T, Mielke S, Almudi I, Nairz K, Froehlich MS, Höckendorff J, Brandt W, Hoehenwarter W, Dohmen RJ, 2016 Phenotypes on demand via switchable target protein degradation in multicellular organisms. Nat Commun 7: 12202. 27447739 10.1038/ncomms12202 PMC4961840

[DONGGAD292235C12] Green AA, Kennaway JR, Hanna AI, Bangham JA, Coen E. 2010 Genetic control of organ shape and tissue polarity. PLoS Biol 8: e1000537. 21085690 10.1371/journal.pbio.1000537 PMC2976718

[DONGGAD292235C13] Haglund K, Stenmark H. 2006 Working out coupled monoubiquitination. Nat Cell Biol 8: 1218–1219. 17077855 10.1038/ncb1106-1218

[DONGGAD292235C14] Haglund K, Di Fiore PP, Dikic I. 2003 Distinct monoubiquitin signals in receptor endocytosis. Trends Biochem Sci 28: 598–603. 14607090 10.1016/j.tibs.2003.09.005

[DONGGAD292235C15] Hicke L, Schubert HL, Hill CP. 2005 Ubiquitin-binding domains. Nat Rev Mol Cell Biol 6: 610–621. 16064137 10.1038/nrm1701

[DONGGAD292235C16] Hoeller D, Dikic I. 2010 Regulation of ubiquitin receptors by coupled monoubiquitination. Subcell Biochem 54: 31–40. 21222271 10.1007/978-1-4419-6676-6_3

[DONGGAD292235C17] Hoeller D, Crosetto N, Blagoev B, Raiborg C, Tikkanen R, Wagner S, Kowanetz K, Breitling R, Mann M, Stenmark H, 2006 Regulation of ubiquitin-binding proteins by monoubiquitination. Nat Cell Biol 8: 163–169. 16429130 10.1038/ncb1354

[DONGGAD292235C18] Husnjak K, Dikic I. 2012 Ubiquitin-binding proteins: decoders of ubiquitin-mediated cellular functions. Annu Rev Biochem 81: 291–322. 22482907 10.1146/annurev-biochem-051810-094654

[DONGGAD292235C19] Johnston LA, Gallant P. 2002 Control of growth and organ size in *Drosophila*. Bioessays 24: 54–64. 11782950 10.1002/bies.10021

[DONGGAD292235C20] Kadrmas JL, Beckerle MC. 2004 The LIM domain: from the cytoskeleton to the nucleus. Nat Rev Mol Cell Biol 5: 920–931. 15520811 10.1038/nrm1499

[DONGGAD292235C21] Kazama T, Ichihashi Y, Murata S, Tsukaya H. 2010 The mechanism of cell cycle arrest front progression explained by a KLUH/CYP78A5-dependent mobile growth factor in developing leaves of *Arabidopsis thaliana*. Plant Cell Physiol 51: 1046–1054. 20395288 10.1093/pcp/pcq051

[DONGGAD292235C22] Kim H, Chen J, Yu X. 2007 Ubiquitin-binding protein RAP80 mediates BRCA1-dependent DNA damage response. Science 316: 1202–1205. 17525342 10.1126/science.1139621

[DONGGAD292235C23] Komander D, Rape M. 2012 The ubiquitin code. Annu Rev Biochem 81: 203–229. 22524316 10.1146/annurev-biochem-060310-170328

[DONGGAD292235C24] Li Y, Zheng L, Corke F, Smith C, Bevan MW. 2008 Control of final seed and organ size by the DA1 gene family in *Arabidopsis thaliana*. Genes Dev 22: 1331–1336. 18483219 10.1101/gad.463608 PMC2377187

[DONGGAD292235C125] Li Z-Y, Li B, Dong A-W. 2012 The *Arabidopsis* transcription factor AtTCP15 regulates endoreduplication by modulating expression of key cell-cycle genes. Mol Plant 5: 270–280. 21992944 10.1093/mp/ssr086

[DONGGAD292235C215] Liu Y, Wang F, Zhang H, He H, Ma L, Deng XW. 2008 Functional characterization of the *Arabidopsis* ubiquitin-specific protease gene family reveals specific role and redundancy of individual members in development. Plant J 55: 844–856. 18485060 10.1111/j.1365-313X.2008.03557.x

[DONGGAD292235C25] Oldham CE, Mohney RP, Miller SLH, Hanes RN, O'Bryan JP. 2002 The ubiquitin-interacting motifs target the endocytic adaptor protein epsin for ubiquitination. Curr Biol 12: 1112–1116. 12121618 10.1016/s0960-9822(02)00900-4

[DONGGAD292235C26] Pan D. 2010 The hippo signaling pathway in development and cancer. Dev Cell 19: 491–505. 20951342 10.1016/j.devcel.2010.09.011 PMC3124840

[DONGGAD292235C27] Peng Y, Chen L, Lu Y, Wu Y, Dumenil J, Zhu Z, Bevan MW, Li Y. 2015 The ubiquitin receptors DA1, DAR1, and DAR2 redundantly regulate endoreduplication by modulating the stability of TCP14/15 in *Arabidopsis*. Plant Cell 27: 649–662. 25757472 10.1105/tpc.114.132274 PMC4558651

[DONGGAD292235C28] Potuschak T, Stary S, Schlögelhofer P, Becker F, Nejinskaia V, Bachmair A. 1998 PRT1 of *Arabidopsis thaliana* encodes a component of the plant N-end rule pathway. Proc Natl Acad Sci 95: 7904–7908. 9653113 10.1073/pnas.95.14.7904 PMC20902

[DONGGAD292235C29] Rawlings ND, Barrett AJ, Bateman A. 2012 MEROPS: the database of proteolytic enzymes, their substrates and inhibitors. Nucleic Acids Res 40: D343–D350. 22086950 10.1093/nar/gkr987 PMC3245014

[DONGGAD292235C30] Reed SI. 2003 Ratchets and clocks: the cell cycle, ubiquitylation and protein turnover. Nat Rev Mol Cell Biol 4: 855–864. 14625536 10.1038/nrm1246

[DONGGAD292235C31] Sato Y, Yoshikawa A, Mimura H, Yamashita M, Yamagata A, Fukai S. 2009 Structural basis for specific recognition of Lys 63-linked polyubiquitin chains by tandem UIMs of RAP80. EMBO J 28: 2461–2468. 19536136 10.1038/emboj.2009.160 PMC2735169

[DONGGAD292235C32] Sluis A, Hake S. 2015 Organogenesis in plants: initiation and elaboration of leaves. Trends Genet 31: 300–306. 26003219 10.1016/j.tig.2015.04.004

[DONGGAD292235C33] Stary S. 2003 PRT1 of *Arabidopsis* is a ubiquitin protein ligase of the plant N-end rule pathway with specificity for aromatic amino-terminal residues. Plant Physiol 133: 1360–1366. 14551326 10.1104/pp.103.029272 PMC281630

[DONGGAD292235C34] Tholander F, Roques B-P, Fournié-Zaluski M-C, Thunnissen MMGM, Haeggström JZ. 2010 Crystal structure of leukotriene A4 hydrolase in complex with kelatorphan, implications for design of zinc metallopeptidase inhibitors. FEBS Lett 584: 3446–3451. 20609366 10.1016/j.febslet.2010.06.044

[DONGGAD292235C35] van der Krogt GNM, Ogink J, Ponsioen B, Jalink K. 2008 A comparison of donor-acceptor pairs for genetically encoded FRET sensors: application to the Epac cAMP sensor as an example. PLoS 3: e1916. 10.1371/journal.pone.0001916 PMC2271053 18382687

[DONGGAD292235C36] Van Wart HE, Birkedal-Hansen H. 1990 The cysteine switch: a principle of regulation of metalloproteinase activity with potential applicability to the entire matrix metalloproteinase gene family. Proc Natl Acad Sci 87: 5578–5582. 2164689 10.1073/pnas.87.14.5578 PMC54368

[DONGGAD292235C37] Varshavsky A. 2011 The N-end rule pathway and regulation by proteolysis. Protein Sci 20: 1298–1345. 21633985 10.1002/pro.666 PMC3189519

[DONGGAD292235C38] Woelk T, Oldrini B, Maspero E, Confalonieri S, Cavallaro E, Di Fiore PP, Polo S. 2006 Molecular mechanisms of coupled monoubiquitination. Nat Cell Biol 8: 1246–1254. 17013377 10.1038/ncb1484

[DONGGAD292235C39] Xia T, Li N, Dumenil J, Li J, Kamenski A, Bevan MW, Gao F, Li Y. 2013 The ubiquitin receptor DA1 interacts with the E3 ubiquitin ligase DA2 to regulate seed and organ size in *Arabidopsis*. Plant Cell 25: 3347–3359. 24045020 10.1105/tpc.113.115063 PMC3809536

[DONGGAD292235C40] Xu F, Zhu C, Çevik V, Johnson K, Liu Y, Sohn K, Jones JD, Holub EB, Li X. 2015 Autoimmunity conferred by chs3-2D relies on CSA1, its adjacent TNL-encoding neighbour. Sci Rep 5: 8792. 25740259 10.1038/srep08792 PMC4350097

