# Supplementary material for: Ubiquitylation activates a peptidase that promotes cleavage and destabilization of its activating E3 ligases and diverse growth regulatory proteins to limit cell proliferation in Arabidopsis
Source: Genes Dev. 2017 Jan 15;31(2):197–208. doi: 10.1101/gad.292235.116 (PMC5322733; doi:10.1101/gad.292235.116)
Supplement: Supplemental Material [file supp_31_2_197__index.html]

Ubiquitylation activates a peptidase that promotes cleavage and destabilization of its activating E3 ligases and diverse growth regulatory proteins to limit cell proliferation in Arabidopsis — Supplemental Material 

# Ubiquitylation activates a peptidase that promotes cleavage and destabilization of its activating E3 ligases and diverse growth regulatory proteins to limit cell proliferation in *Arabidopsis*

## Supplemental Material

undefined

- Supplemental\_Information.docx
