# Supplementary material for: Ubiquitylation activates a peptidase that promotes cleavage and destabilization of its activating E3 ligases and diverse growth regulatory proteins to limit cell proliferation in Arabidopsis
Source: Genes Dev. 2017 Jan 15;31(2):197–208. doi: 10.1101/gad.292235.116 (PMC5322733; doi:10.1101/gad.292235.116)
Supplement: Supplemental Material [file supp_gad.292235.116_Supplemental_Information.docx]

**SUPPLEMENTAL MATERIAL**

**Methods**

**Plasmid Construction**

The coding regions of genes were isolated from Arabidopsis Col-0 seedling cDNA using specific primers (Table S1) and Phusion Hi Fidelity DNA polymerase (NEB, Hitchin, UK). The coding regions of *DA1* (At1g19270), *BB* (At3g63530), *DA2* (At1g78420) and *BBR* (At3g19910) were cloned into pETnT (adapted from Novagen pET 24a) to create FLAG- and HIS- tagged proteins for expression in *E. coli* from the T7 promoter. Cloning used BamH1/Xho1 or EcoR1/Xho1 restriction sites. The coding region of DA1 containing UIM1 and UIM2 (amino acids 1-167) was amplified and cloned into the BamH1 and Xho1 sites of pGEX4T2 to (GE Healthcare, Little Chalfont, UK) to create GST UIM1+2. Mutant UIM1, UIM2 and UIM1+2 sequences were generated by overlap PCR using primers described in Table S1. Cloning into the binary vectors pEarley-gate (Earley et al., 2006), PW1211/66 (vectors kindly provided by P. Wigge), or pEAQ (Peyret and Lomonossoff, 2013) for expression *in planta* from the 35S promoter used Gateway cloning via the entry vector pENTR Topo D (Invitrogen). Topo D entry clones were transferred into PW2122 (C- terminal 3xFLAG), PW1266 (N- terminal 3xFLAG), pEarley-gate 103 (GFP-His), pEarley-gate 201 (HA), pEAQ-HT-DEST3 (C term HIS tag), and pEAQ-HT-DEST2 (N term HIS tag used for FLIM-FRET reporter construction). Recombination used LR Clonase for 4 h and transformation into DH5α, Mach1-TI or Top10 competent cells. Transformants were selected on 25 μg/ml kanamycin LB plates. FLIM-FRET constructs were based on the Epac sensor (van der Krogt et al., 2008) containing eGFP and mCherry. Briefly, the epac sequences between eGFP and mCherry were removed using EcoRV and Nhe1, and BB was amplified from a topo D clone using primers for Eco Rv and Nhe1 cloning. The eGFP-BB-mCherry coding region was cloned into Topo D and then into pEAQ-HT-DEST2 for expression *in planta*. *UBP15* (At1g17110), *TCP5* (At5g60970), *TCP14* (At3g47620), *TCP15* (At1g69690) and *TCP22* (At1g72010) were amplified using primers shown in Table S1, and the PCR products were cloned into the binary vector pEarley3FLAG (constructed from pEARley103) using Infusion cloning (Clontech). Briefly, pEarley3FLAG was linearized by Xho1 digestion, and PCR products without stop codons were inserted to generate the construction UBP15-3FLAG, TCP5 -3FLAG, TCP14 -3FLAG, TCP15 -3FLAG and TCP22 -3FLAG. To generate C- terminal 3FLAG fusions, pEARley3FLAG was linearized by Xba1 digestion and PCR products with stop codons were inserted into to generate 3FLAG-UBP15, 3FLAG -TCP5, 3FLAG -TCP14, 3FLAG - TCP15 and 3FLAG -TCP22 fusions. *DA1* and mutant versions were amplified and the PCR product cloned into the binary vector pEARley3HA (constructed from from pEarley103) using Infusion reactions. pEarley3HA was linearized by Xba1 digestion, PCR products with stop codons were inserted into the vector pEarley3HA to generate 3HA-DA1 gene fusions. The Arabidopsis polyubiquitin gene *UBC10* (At4g05320) was cloned into pEarley3HA to generate an N-terminal 3HA fusion.

*In vitro* mutagenesis was carried out with GeneArt Site-directed mutagenesis (Life Technologies, Paisley, UK) using a Topo D entry clone as template. Mutagenic Primers are described in Table S1. The *in vivo* expression constructs *35S:Myc-DA1* and *35S:GFP-DA2* were described previously (Xia et al., 2013). The coding region of TR-TUBE (Yoshida *et al* 2015) was amplified from plasmid FLAG-TR-TUBE in vector pcDNA-FLAG using primers with Xho1 and EcoR1 sites, and clones into pETnT to create FLAG fusion proteins for expression in *E. coli.* PRT1 was cloned with a 5’ TEV recognition sequence into the pDONR201 (Invitrogen) vector, using primers shown in Table S1, followed by an LR reaction into the vector pVP16 to make an 8xHIS:MBP:PRT1 fusion protein.

**Construction of BB proteins for *in vivo* cleavage and degradation.**

The coding sequence of BB (AY-GG) were made by GeneArt® Site-Directed Mutagenesis PLUS Kit (Invitrogen, A14604) from the TOPOD-BB vector. Linearization of pEARley103 was carried out by FastDigest XhoI and FastDigest XbaI (Thermo Scientific FD0649 and FD0684) and ligated with the gsGreen coding sequence removed the stop codon with In-Fusion® HD Cloning Kit (Clontech, PT5162). The gsGreen coding sequence was amplified from the *35S::gsGreen-BB* plasmid with primers (gsGreen in-pEARley103 Xho1 F and gsGreen in-pEARley103 Xba1 R) by Q5® High-Fidelity DNA Polymerase (NEB, M0491). The construct composed of pEARley103 and the gsGreen coding sequence was named pEARley-gsGreen. This was linearized by FastDigest XbaI (Thermo Scientific FD0684) and ligated with BB, BB AY-GG and BB (C215A H217A) coding sequence with In-Fusion® HD Cloning Kit (Clontech, PT5162) separately. The BB coding sequence was amplified from *35S::gsGreen-BB*, *TOPOD-BB AY-GG* and *35S::gsGreen-BB (C215A H217A)* plasmids with primers (EOD1 ingsGreen F and EOD1 inpEARley103 Xba1 R) by Q5® High-Fidelity DNA Polymerase (NEB M0491). N-terminal fusions of ubiquitin with BB were made using the Ubiquitin Fusion Technique (Baker 1996) as described (Naumann et al 2016). First, Ubiquitin was amplified with primers ss_bridge_attB1-UBQ and asY/G_EOD1 (according to the N- terminus) and in parallel X-EOD1-HA (with or without stop codon) was amplified using the primers ss_UBQ_Y/G_EOD1 and as_EOD1_HAT. M61-EOD1-HA_stop was cloned using the primer ss_attB1_M_EOD1 as sense primer. The HA-tag was cloned with or without stop codon using the primers ss_HAT_EOD1 and as_EOD1_HA_stop/os. The Fragments were fused together using the primers ss_UBQ-attB1/ss_attB1_M_EOD1 and the desired antisense primer as_EOD1_HA_Stop/os. All primer sequences are listed in the Table below. The constructs were cloned into pDONR201 followed by a LR- reaction into pOLENTE (for *in vitro* stability) or pAM-PAT-Luciferase (for protoplast transformation).

***In vitro* degradation**

Constructs coding for X61-EOD1-HA with different N-termini (X = Y, G, MY) were expressed in a cell free expression system (TnT® Quick Coupled Transcription/Translation System, Promega L4610) under the control of the T7 promotor. 250 ng were used for a total reaction volume of 12.5 μl (quarter reaction) (Naumann et al 2016). Samples were incubated at 30^0^C for 30 min, with or without 100 μM MG132 for proteasome inhibitor treatment. Translation inhibitor Cycloheximide (CHX) was then added to the reaction to a final concentration of 200μM. Samples (5μl) were taken at time points 0 and 90 minutes after commencing treatment, directly resuspended in 8μl 5x SDS loading dye + 24μl H2O and frozen in liquid nitrogen to prevent degradation. After the experiment was finished samples were heated to 65 degrees for 15min and loaded on a 12% SDS gel (BioRad) using a 10x comb. Following gel separation samples were blotted on a PVDF membrane (Millipore) using a semi-dry blotting chamber (BioRad). After blotting the membrane was blocked using 5% milk in TBST. Immunoblotting used 1.AK α-HA 1:1000 (COVANCE, MMS-101R) in 3% milk, followed by 3 times washing with TBST and incubation with 2. AK α-mouse-HRP (Thermo, sc-200431430; 3% milk, 1:5000). Blots were detected with Pico-Substrate for 90 sec. Blots were stained afterwards with CBB.

***In vivo* stability in *Arabidopsis* mesophyll protoplasts**

Constructs encoding X61-EOD1-HA-Luciferase with different N-termini (X = Y, G) were expressed in mesophyll protoplasts from Col-0 and *prt1* plants under the control of the 35S promotor. Protoplasts were isolated as described below, and transfected with 4 μg/100μL protoplasts with X-61-EOD1:HA:Luciferase (X = Y, G) and 1 μg/100 μL protoplasts pUBC::GUS as control. After incubation over night at 21 degrees, GUS activity was measured by adding 10 μL protoplast solution to 190 μL GUS-extraction buffer (50 mM NaPO4 pH 7; 10 mM EDTA, 0.1% Triton, 0.1 % Tween)) supplied with 1 mM MUGlcU. Measurement was done in a Tecan MX 1000 at 37°C, in 30 cycles (2 min). Luciferase activity was measured by adding D-luciferin in a 1:100 dilution to the protoplasts, incubation for 10 min in the dark. Luminescence was measured for 5 sec 5 times in an Tecan M200 Pro and the mean was normalized to GUS. Normalized value for G61-EOD1- HA-Luciferase was taken as 100% and stability of Y61-EOD1-HA-Luciferase was calculated .

**Expression of proteins in *E. coli* and their purification**

Recombinant proteins were expressed from the peTnT vector as FLAG or HIS fusions, or from pGEX4T2 as GST fusions. Cultures of BL21 were treated with 1mM IPTG to induce expression for 3 h at 28^o^C. The cultures were then centrifuged and the bacterial pellet re-suspended at 4^o^C in TGH buffer (50mM HEPES-KOH pH 7.5, 150mM NaCl, 1% Triton X-100, 10% glycerol, 1mM DTT) and 1 EDTA-free protease inhibitor tablet per 50 ml buffer. DTT was not used when purifying FLAG fusion proteins. The bacterial suspension was sonicated on ice with 4 bursts of 10 sec each before centrifugation at 12,000 x g for 20 min. The cleared sonicate was incubated on ice with glutathione-Sepharose (GE Life Sciences, Amersham, UK) to purify GST fusion proteins, with magnetic beads coupled to anti-FLAG (Sigma-Aldrich, Gillingham, UK), or with HIS-Select Nickel Affinity Gel (Sigma-Aldrich). Bound proteins were washed in TGH buffer on ice, released from the affinity resin (GST fusion proteins by 50mM Tris-glycine pH 8.0, 10 mM reduced glutathione; FLAG fusion proteins by FLAG peptide; HIS fusion proteins by 200 mM imidazole), quantified using Bradford reagent, frozen in LN_2_ and stored at -70^o^C.

**Ubiquitin binding analyses**

GST-UIM1+2 fusion proteins and mutant variants were expressed in *E. coli*, bound onto glutathione agarose beads, and washed in TGH buffer. The beads were incubated with 10 μg ubiquitin in 100 μl TGH buffer at 4^o^C for 2 h, after which the beads were washed four times with TGH and added to SDS sample buffer. Eluted proteins were electrophoresed on 20% SDS-PAGE and immunoblotted with mouse monoclonal anti- ubiquitin (Boston Biochem) at 1:1000 dilution.

**Protoplast Transfection**

Protoplasts were isolated from *da1-kodar1* or *da1-kodar1da2* plants to reduce endogenous DA1, DAR1 and DA2 activity. Leaf mesophyll protoplasts were used for in vivo DA1 peptidase assays. The lower epidermis of leaves of 3-4 week old plants grown on GM medium was stripped using Scotch magic tape (Wu et al., 2009) and the remaining leaf tissue incubated with 1.5% cellulase Onozuka R10 (Yakult Pharmaceutical Industry, Tokyo, Japan) and 0.4% macerozyme (Duchefa Biochemie, Haarlem, The Netherlands) in W5 medium (20 mM KCl, 0.4M mannitol, 20 mM MES pH5.7), vacuum infiltrated for 3 min, incubated at room temperature with gentle shaking for 3 h, filtered through 75 μm nylon mesh, and centrifuged at 100 x g for 3 min. Protoplasts were gently re-suspended in cold W5 medium, incubated on ice for 30 min, centrifuged and resuspended in MMg solution (0.4M mannitol, 15mM MgCl_2_, 4 mM MES pH5.7) to 2 x 10^5^ protoplasts/ml. DNA (up to 20 μg in 20 μl) was added to 100 μl protoplasts, and 220 μl PEG/Ca solution was added (40% PEG 3350 in 0.2M mannitol, 100 mM Ca(NO_3_)_2_, pH 5.7). The protoplasts were incubated at room temperature for 30 min, diluted with 400 μl W5 solution (154 mM NaCl, 125 mM CaCl_2_, 5 mM KCl, 2 mM MES pH 5.7), centrifuged, the supernatant was completely removed, and the transfected protoplasts were resuspended in 100 μl W5. This was added to 400 μl W5 in a 24 well microtitre plate and incubated at 20^O^C overnight in constant low light before protein extraction. Where used, MG132 was added to a final concentration of 50 μg/ml. For FRET-FLIM experiments, root protoplasts were used to reduce chlorophyll fluorescence. Roots from plate grown 3-4 week plants were harvested, vacuum infiltrated with W5 medium containing 1.5% cellulase (Yakult), 1% cellulysin (Merck Millipore, Darmstadt, Germany) and 0.1% pectolyase Y-23 (Duchefa), finely chopped, and purified and transfected as described above. Root protoplasts were centrifuged at 1000 x g for 3 min. After incubation, protoplasts were collected by centrifugation at 100x g for 2.5 minutes, and processed as described below.

**Arabidopsis Protoplast and *Nicotiana benthamiana* Transient Gene Expression**

For DA1 cleavage assays, Arabidopsis *da1kodar1* leaf or root protoplasts were transfected with plasmids as described above. Proteins were extracted and analysed by SDS-PAGE and immunoblotting. To detect ubiquitylation of DA1, *35S:Myc-DA1* or *35S:Myc-DA1*(*UIM1+2*) were co-expressed with either *35S:GFP-DA2* or empty vector pMDC43 in *N. benthamiana* leaves. Total protein extract was immunoprecipitated with Agarose-conjugated Myc-Tag Mouse mAb (Abmart) and immunoblotted.

**Stable Transformation of Arabidopsis**

Arabidopsis transformation was carried out by floral dip in *Agrobacterium tumefaciens* GV3101 containing binary vector constructs described above. Stable transformants were selected on BASTA and homozygous single copy insertion lines identified.

**Detection of DA1 ubiquitination *in vivo***

Three- week old plants of *35S*::*GFP-DA1* and *35S*::*GFP* T3 transgenic plants grown in MS medium on plates were transferred into liquid MS media with 50 µM Mg132 (Sigma Aldrich M7449) for 6 hours. They were gently shaken (85rpm, 16 hours light (20^o^C) and 8 hours dark (18^o^C)). Plantlets were harvested, dried on paper towels and frozen and ground in LN_2_. Lysis buffer (Yoshida, et al. 2015) (10 mM Tris/HCl pH 7.5; 150 mM NaCl; 0.5 mM EDTA; 10% Glycerol; 0.5% NP-40; EDTA-free Protease Inhibitor Cocktail (Roche, 04693159001); 24 µM TR-TUBE protein and 50 µM Mg-132 (Sigma Aldrich M7449)) was added at 2 mL/g to the tissue powder, mixed well, and keep in ice for 10min. Samples were centrifuged by 5,000 rpm for 20 min at 4 ^o^C, and the supernatant filtered with Miracloth into a precooled tube. 1.5 volumes of precooled dilution buffer was added to the filtrate (10 mM Tris/HCl pH 7.5; 150 mM NaCl; 0.5 mM EDTA; 10% Glycerol; Protease Inhibitor Cocktail (Roche, 04693159001); 24 µM TR-TUBE and 50 µM Mg132(Sigma Aldrich M7449). GFP-Trap-A beads (Chromotek, gta-10) were prepared according to the manufacturer’s recommendation, and GFP-Trap purification was performed using the GFP-Trap®_A for Immunoprecipitation of GFP-Fusion Proteins protocol. The purified samples were loaded into 4-20% precast SDS-polyacrylamide gels (RunBlue NXG02012, NXG01227, NXG42027) and Western blotted with anti-GFP (Miltenyi Biotec, 130-091-833), anti-Plant Ubiquitin (Bethyl Laboratories, A300-318A), and Anti-α-Tubulin (Sigma, T5168) separately. TR-TUBE protein was expressed from the pETNT vector in BL21(DE3) pLysS cell (Invitrogen, C606003), and purified using Anti-FLAG® M2 Magnetic Beads (for FLAG-tag, Sigma-Aldrich M8823.36).

**Detection of BB cleavage *in vivo***

Eight-day seedlings of *eod1-2*, *pEOD1::gsGreen-EOD1*, *pEOD1::gsGreen-EOD1 (C215A H217A)* and *35S*::*GFP* plants were grown in liquid MS media with 85rpm shaking and then treated with 50 µM Mg132 (Sigma Aldrich M7449) for 6 hours. The seedling were harvested, dried and ground in LN_2_. Lysis buffer (Yoshida, et al. 2015) (10 mM Tris/HCl pH 7.5; 150 mM NaCl; 0.5 mM EDTA; 10% Glycerol; 0.5% NP-40; EDTA-free Protease Inhibitor Cocktail (Roche, 04693159001); 24 µM TR-TUBE and with or without 50 µM Mg132 (Sigma Aldrich M7449)) was added at 2 mL/g tissue powder, mixed well, and keep on ice for 10min. Samples were centrifuged by 5,000 rpm for 20 min at 4 ^o^C, and the supernatant filtered through Miracloth into a precooled tube. 1.5 volumes of precooled dilution buffer (10 mM Tris/HCl pH 7.5; 150 mM NaCl; 0.5 mM EDTA; 10% Glycerol; EDTA-free Protease Inhibitor Cocktail (Roche, 04693159001); 24 µM TR-TUBE, with or without 50 µM Mg132 (Sigma Aldrich M7449)) was added to the filtrate. GFP-Trap-A bead purification was as described above. Samples were loaded into 4-20% precast SDS-polyacrylamide gels (RunBlue NXG02012, NXG01227, NXG42027) and then performed by Western blotting with anti-GFP (Abcam, ab290).

**Protein Extraction, Immunoprecipitation and Immunoblotting**

Protoplasts were harvested by centrifugation and resuspended in 400 μl 100 mM Tris-HCl, pH7.5, 150 mM NaCl, 1% Triton X-100, 5% glycerol, and 1 EDTA-free protease inhibitor tablet (Roche Life Science, Burgess Hill, UK) per 50 ml buffer. The protoplasts were vortexed and sonicated briefly (2 x 5 sec) on ice, and then centrifuged at 12,000 x g for 5 min. The supernatant was either directly used for immunopurification or an equal volume of SDS-SB added, heated at 96^O^C for 10min, then frozen at -70^O^C before SDS-PAGE. Leaf material from Agrobacterium-inoculated *N. benthamiana* plants was extracted and processed in the same way using 1 ml of extraction buffer.

Proteins were immunopurified on FLAG- or HA- magnetic beads (Sigma-Aldrich) or GFPTrap-A (Chromotek) according to the manufacturer’s technical bulletin. According to the proteins and reactions, either protoplast extract buffer (see above), or 50mM Tris-HCl pH7.5, 150 mM NaCl, 5 mM EDTA, 1% (v/v) Triton X100, 2 mM NaF, 2 mM Na_3_VO_4_, and 1 tablet of protease inhibitor tablet (Roche) per 50 ml (Zhou et al., 2014) for *in vivo* ubiquitylated proteins. Proteins were eluted with FLAG- or HA- peptide, or SDS sample buffer. Protein samples in SDS sample buffer were electrophoresed on precast 12%, 20% or 4-20% gradient SDS polyacrylamide gels (RunBlue, Expedeon Ltd, Cambridge, UK), transferred to PVDF membranes (Roche Diagnostics, Burgess Hill, UK) and immunoblotted. PVDF membranes were washed for 10 min in 50 ml PBS after transfer, then treated with blocking solution (5% w/v milk powder, 0.1% v/v Tween-20 in PBS) for an h. Primary antibodies were diluted in blocking solution and incubated with the membrane for 1 h before 5 washes with PBST (PBS with 0.1% v/v Tween-20) at room temperature. Washed membranes were then treated with FEMTO Max peroxidase substrate (Fisher Scientific, Loughborough, UK) for 5 min before exposure to X-ray film. The antibodies used were: FLAG M2-HRP mouse monoclonal, HIS6-HRP mouse monoclonal, HA-HRP mouse monoclonal (Sigma-Aldrich), GST-HRP, goat-HRP, Mouse-HRP (Santa Cruz Biotech, Insight Biotechnology Wembley, UK), Ubiquitin mouse monoclonal (Boston Biochem), and GFP-HRP (Miltenyi Biotech, Bisley, UK).

***In vitro* ubiquitylation assays**

Reactions were carried out in 30 μl final volume at 30^o^C for 2 h using 100ng E1 (human UBE1, Boston Biochem, Boston, USA), 500 ng E2 (either purified GST-UBC10 or human Ubch5b from Boston Biochem), and 200ng of E3 (EOD1, DA2 or BBR expressed as FLAG-fusion proteins from peTnT (adapted from Takara/Clontech, St Germain-en-Laye, France). The reaction buffer contained 50mM Tris-HCl pH 7.4, 5mM MgCl_2_, 2mM ATP, 2mM DTT, and 10 μg/ml ubiquitin (human recombinant, Boston Biochem). FLAG-DA1 and mutant versions were used at 200ng per reaction. Reactions were terminated by incubation at 4^o^C for 10 min.

**SPOT Assays**

An array of synthetic 17-mer peptides was synthesized according to Klecker and Dissmeyer (in press). The peptide backbone sequence was an N-recognin test substrate XZ-FSTDTGPGHLQKKSG with alternating N-terminal amino acids, XZ = LK, KK, MK, FK, PK, SK, TK, WK, YK, VK. SPOT array peptide synthesis was performed with a ResPep SL pipetting robot (INTAVIS) on a PEG-derivatized cellulose membrane (INTAVIS) (Gausepohl et al 2002) from the corresponding N-α-Fmoc- amino acids (INTAVIS). The membrane was incubated overnight in blocking solution (20 mM Tris-HCl, pH 7.4; 135 mM NaCl; 0.1% (v/v) Tween-20; 5 mM DTT; 10% (w/v) skim milk powder) at 4°C. The next day, purified recombinant PRT1 was added to binding buffer (20 mM Tris-HCl, pH 7.4; 135 mM NaCl; 0.1% (v/v) Tween-20; 5 mM DTT; 10 mM maltose; 0. 5% (w/v) skim milk powder) to a final concentration of 36 nM. The mixture was incubated under rotation for 15 min before transfer to the membrane. After 2 h of membrane binding under agitation at room temperature, the array was washed three times for 10 min with binding buffer lacking milk powder. Subsequently, membrane- borne protein was subjected to electrotransfer to a PVDF membrane (0.6 mA per cm2 of membrane, 30 min) 14. The protein was probed by immunodetection using Anti-His antibody (GE Healthcare; No. 27-4710-01) and goat anti-mouse HRP conjugated secondary antibody (Pierce; No. 31437).

**Lifetime-Imaging Microscopy**

Root protoplasts were isolated as described above, transfected and harvested after 18 h. Protoplasts were re-suspended in W5 containing 0.1% agarose to reduce movement, and imaged as described in Experimental Procedures. Fluorescence Lifetime IMaging-Förster Resonance Energy Transfer (FLIM-FRET) experiments where carried out on a LaVision Biotec TriM scope II multi-photon microscope using a 63x/1.2NA water-immersion objective and a 16-channel TCSPC detector. Excitation at 880 nm was provided by an 80 MHz pulsed-fs laser (Coherent Vision II Ti:Sapphire). Emission wavelengths were: GFP - 500-550 nm and RFP – 590-650 nm. Measurements of fluorescence lifetimes were obtained over a period of 11.2 ns with timepoints every 80 ps. Time-constants (τ) were calculated by least-squares fitting of the exponential fluorescence decays using ImSpectorPro software. The images in the top panel of Figure 4 show the spatial variation of τ over the cells in a pixel-by-pixel fashion with a Look-Up Table (LUT) that represents short lifetimes with cool colours and longer lifetimes with warmer colours. For analysis, 3 representative 10 μm x 10 μm square Regions-Of-Interest (ROIs) were chosen in each cell, and the average τ in each ROI calculated. The results in the middle panel of Figure 4 represent 13 cells for each type of transfection.

**Mass Spectrometry of Ubiquitylated DA1**

FLAG-DA1, FLAG-DA1 (R358K) and FLAG-DA1- (4K-4R) were expressed in *E.coli* BL21 cells and purified as described above. A large-scale in vitro ubiquitylation reaction using DA2-HIS was carried out to prepare 2-4 ug of ubiquitylated DA1 and variants. These we purified on FLAG magnetic beads, electrophoresed on SDS-PAGE, and visualised by light staining with Instant Blue. Bands between 100-130 kDa were carefully excised, washed, reduced and alkylated, and treated with trypsin according to standard procedures based on (Shevchenko et al., 2006). Peptides were extracted with 5% formic acid/50% acetonitrile, dried down, and re-dissolved in 0.1% TFA. For LC-MS/MS analysis, a sample aliquot was applied via a nanoAcquity^TM^ (Waters Ltd, Manchester, UK) UPLC^TM^-system running at a flow rate of 250 nL min^-1^ to an LTQ-Orbitrap™ mass spectrometer (Thermo Fisher, Waltham, MA). Peptides were trapped using a pre-column (Symmetry ^®^ C18, 5µm, 180 µm x 20 mm, Waters Ltd) which was then switched in-line to an analytical column (BEH C18,1.7 µm, 75 µm x 250 mm, Waters Ltd) for separation. Peptides were eluted with a gradient of 3-37% acetonitrile in water/0.1% formic acid at a rate of 0.5% min^-1^. The column was connected to a 10 µm SilicaTip™ nanospray emitter (New Objective, Woburn, MA, USA) attached to a nanospray interface (Proxeon, Odense, Denmark) for infusion into the mass spectrometer. The mass spectrometer was operated in positive ion mode at a capillary temperature of 200 °C. The source voltage and focusing voltages were tuned for the transmission of MRFA peptide (m/z 524) (Sigma-Aldrich, St. Louis, MO). Data dependent analysis was carried out in oribtrap-IT parallel mode using CID fragmentation of the 5 most abundant ions in each cycle. The orbitrap was run with a resolution of 30,000 over the MS range from m/z 350 to m/z 1800 and an MS target of 10^6^ and 1 s maximum scan time. Collision energy was 35, and an isolation width of 2 was used. Only mono-isotopic 2+ and 3+ charged precursors were selected for MS2. The MS2 was triggered by a minimal signal of 1000 with an AGC target of 3x10^4^ ions and 150 ms scan time using the chromatography function for peak apex detection. Dynamic exclusion was set to 1 count and 30 s exclusion with an exclusion mass window of ±20 ppm. MS scans were saved in profile mode while MSMS scans were saved in centroid mode.

Raw files from the orbitrap were processed with MaxQuant version 1.3.0.5 (Cox and Mann, 2008) (<http://maxquant.org>) to generate re-calibrated peaklist-files which were used for database searches using an in-house Mascot® 2.4 Server (Matrix Science Limited, London, UK). The searches were performed on the TAIR10_pep_20101214 fasta database ([www.arabidopsis.org](http://www.arabidopsis.org)) and on a common contaminants database using trypsin/P with 2 missed cleavages, carbamidomethylation (C) as fixed, and oxidation (M) and acetylation (protein N-terminus) as variable modifications. Mass tolerances were 6 ppm for precursor ions and 0.6 Da for fragment ions. Mascot search results were imported and evaluated in Scaffold 3.6.4 (proteomsoftware.com, Portland, OR, USA) with thresholds of 99% and 95% for proteins and peptides.

**Edman Sequencing of cleaved EOD1**

Cleaved EOD1-HIS was generated in a large- scale cleavage reaction using approximately 10 μg FLAG-DA1 that had been ubiquitylated in a separate reaction and purified on FLAG-resin. Approximately 20 μg of FLAG-EOD-HIS was incubated with FLAG-DA1^Ub^ for 4h at 30^o^C, and the reaction purified on FLAG magnetic beads to remove un-cleaved EOD1 and DA1^Ub^. Bound protein was eluted with imidazole, treated with SDS- sample buffer and electrophoresed on SDS-PAGE. The gel was transferred to PVDF membrane and briefly stained with Instant Blue (Expedion) to reveal the 35 kDa cleaved product. This region of the membrane was excised, rinsed in methanol and air- dried. The protein was N-terminal sequenced using a Shimadzu PPSQ-31B protein sequencer. Edman degradation was carried out for 6 cycles (Figure S5).

**Gene Expression Analysis during Leaf Development**

For early time- points (5 to 13 DAS), whole seedlings were harvested in an excess of RNAlater solution (Ambion) and, after overnight storage at 4^◦^C, dissected under a binocular microscope on a cooling plate with surgical needles. For later time- points, leaves were directly dissected from the plants. The isolated leaves were transferred to a 2ml Eppendorf tube and frozen in LN_2_. Leaf material was ground with a Retsch machine and 4-mm metal balls. RNA extractions were performed with TRIzol reagent (Invitrogen). To eliminate the residual genomic DNA present in the preparation, RNA was treated by RQ1 RNAse-free DNase according to the manufacturer's instructions (<http://www.promega.com>) and purified with the RNeasy Mini kit (<http://www.qiagen.com>). Complementary DNA was made with the iScript cDNA Synthesis kit from Biorad (<http://www.bio-rad.com>) according to the manufacturer's instructions. Q-RT-PCR was performed on a LightCycler 480 (<http://www.roche.com>) in 384-well plates with LightCycler 480 SYBR Green I Master (Roche) according to the manufacturer's instructions. The expression data was normalized using three housekeeping genes (AT1G13320, AT2G32170 and AT2G28390).

Primers used for gene expression analyses are shown in Table S2.

**Growth Measurements**

The width of leaf one of plate-grown Arabidopsis seedlings (n=8) were measured from scaled images. Growth curves were obtained using the sigmoidal function of Sigmaplot 13.

**Supplemental References**

Baker R. Protein expression using ubiquitin fusion and cleavage. Curr Opin Biotechnol *7,* 541-546 (1996).

Cox, J., and Mann, M. (2008). MaxQuant enables high peptide identification rates, individualized p.p.b.-range mass accuracies and proteome-wide protein quantification. Nat Biotechnol *26*, 1367–1372.

Earley, K.W., Haag, J.R., Pontes, O., Opper, K., Juehne, T., Song, K., and Pikaard, C.S. (2006). Gateway-compatible vectors for plant functional genomics and proteomics. The Plant Journal *45*, 616–629.

Gausepohl H, Behn C. Automated Synthesis of Solid-Phase Bound Peptides. In: Springer Lab Manual - Peptide Arrays on Membrane Supports. Springer-Verlag (2002).

Klecker M, Dissmeyer N. Peptide arrays for binding studies of E3 ubiquitin ligases. Methods Mol Biol, (in press).

Naumann, C., Mot, A.C., and Dissmeyer, N. (2016). Generation of Artificial N-end Rule Substrate Proteins In Vivo and In Vitro. Methods Mol. Biol. *1450*, 55–83.

Peyret, H., and Lomonossoff, G.P. (2013). The pEAQ vector series: the easy and quick way to produce recombinant proteins in plants. Plant Mol Biol *83*, 51–58.

Shevchenko, A., Tomas, H., Havlis, J., Olsen, J.V., and Mann, M. (2006). In-gel digestion for mass spectrometric characterization of proteins and proteomes. Nat Protoc *1*, 2856–2860.

van der Krogt, G.N.M., Ogink, J., Ponsioen, B., and Jalink, K. (2008). A Comparison of Donor-Acceptor Pairs for Genetically Encoded FRET Sensors: Application to the Epac cAMP Sensor as an Example. PLoS ONE *3*, e1916.

Wu, F.-H., Shen, S.-C., Lee, L.-Y., Lee, S.-H., Chan, M.-T., and Lin, C.-S. (2009). Tape-Arabidopsis Sandwich - a simpler Arabidopsis protoplast isolation method. Plant Methods *5*, 16.

Yoshida, Y., Y. Saeki, A. Murakami, J. Kawawaki, H. Tsuchiya, H. Yoshihara, M. Shindo and K. Tanaka (2015). "A comprehensive method for detecting ubiquitinated substrates using TR-TUBE." Proc Natl Acad Sci U S A 112(15): 4630-4635.

Zhou, J., He, P., and Shan, L. (2014). Ubiquitination of Plant Immune Receptors. In Methods in Molecular Biology, (New York, NY: Springer New York), pp. 219–231.

**Supplementary Figures**

**
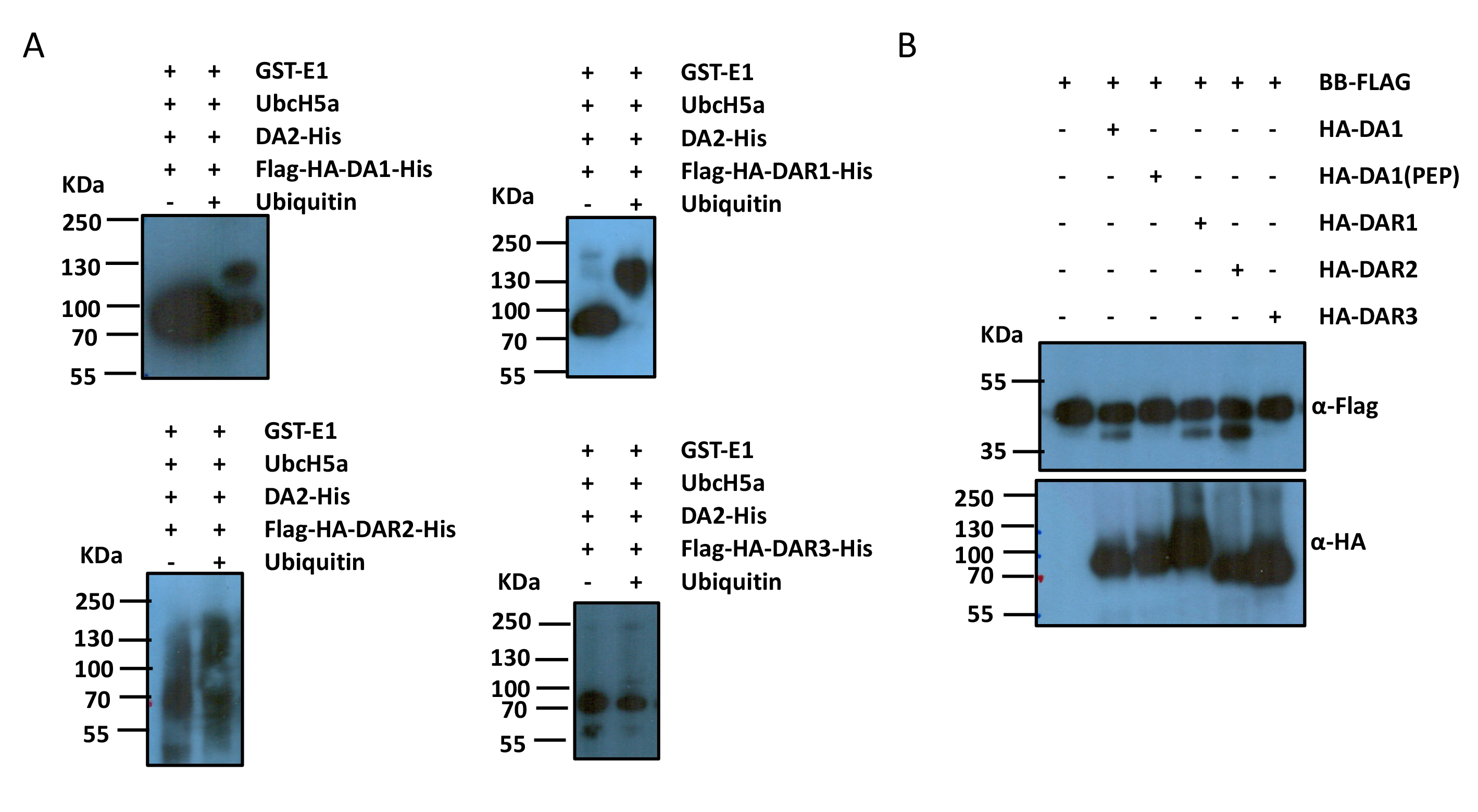
**

**Supplemental Fig.1. Ubiquitylation of DA family members by the E3 ligase DA2, and the cleavage of BB-FLAG by DA family members.**

(A) western blot of FLAG-DA1, FLAG-DAR1, FLAG-DAR2 and FLAG-DAR3 ubiquitylation by DA2-HIS *in vitro*. Anti-FLAG antibody revealed that FLAG-DAR3 was not ubiquitylated by DA2, while the other family members were.

(B) western blot of BB-FLAG cleavage in protoplasts by HA-DA1, HA-DAR1, HA-DAR2 and HA-DAR3. HA-DAR3 did not cleave BB-FLAG.

**Supplemental Fig. 2. Ubiquitylation patterns of DA1**

Each panel shows the outputs of experiments determining ubiquitylated amino acids on wild-type DA1 protein. The outputs are from Scaffold 3.6.4 and show the protein sequence of DA1, with trypsinised peptides identified by yellow highlights. Modified amino acids are shown in green highlights. Lysines modified by ubiquitylation are identified by hatched boxes or circles in each panel.

Panel A. Four analyses of ubiquitylated wild-type DA1 are shown, with peptide coverage of 49-59%. The red hatched boxes show lysine residues that were consistently modified in each of the four analyses. The purple- and blue-hatched boxes show modified lysines found in three and two analyses respectively. Blue circles identify modified lysines identified in a single sample.

Panel B. Two analyses of ubiquitylated DA1(4K4R) are shown, with peptide coverage of 59-60%. Modified lysines identified in both analyses are circled in red, and the position of original lysines mutated to arginines are shown by black circles.

Panel C. A single analysis of ubiquitylated DA(R358K) is shown, with peptide coverage of 45%. Modified lysines are circled in black, and lysine 358 is circled in red.


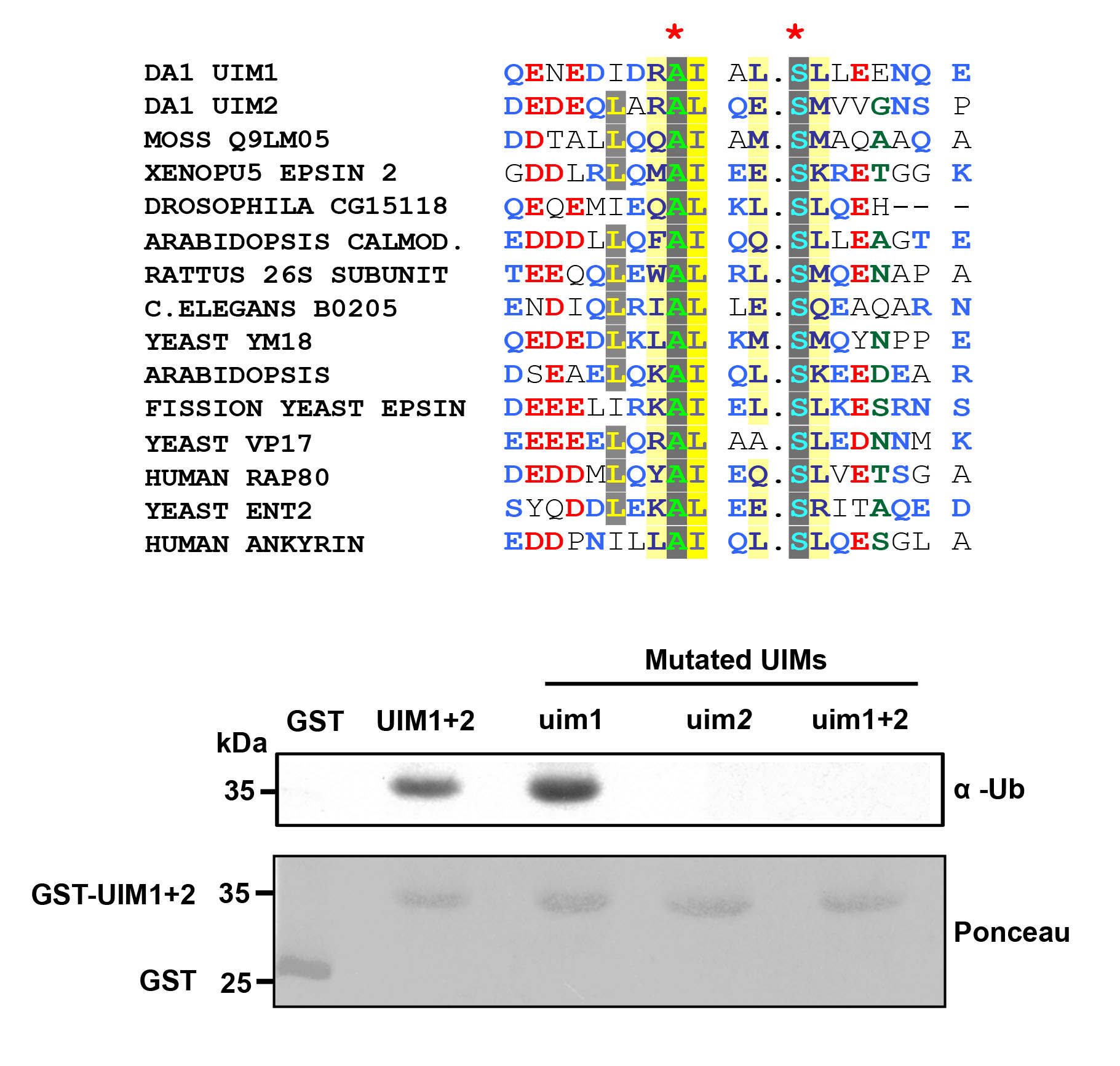


**Supplemental Fig. 3. Binding of Ubiquitin to the DA1 Ubiquitin Interaction Motifs**

The top panel shows an alignment of UIM1 and UIM2 from DA1 with related UIMs from diverse organisms. Conserved A and S residues identified by an asterisk were mutated to G in each UIM separately and together in a GST-UIM1+2 fusion protein. The central panel is a western blot using anti- Ubiquitin to show pull-down assays of ubiquitin binding. The mutations in conserved A and S residues strongly reduced binding to Ub of UIM2. The lower panel is a loading control.


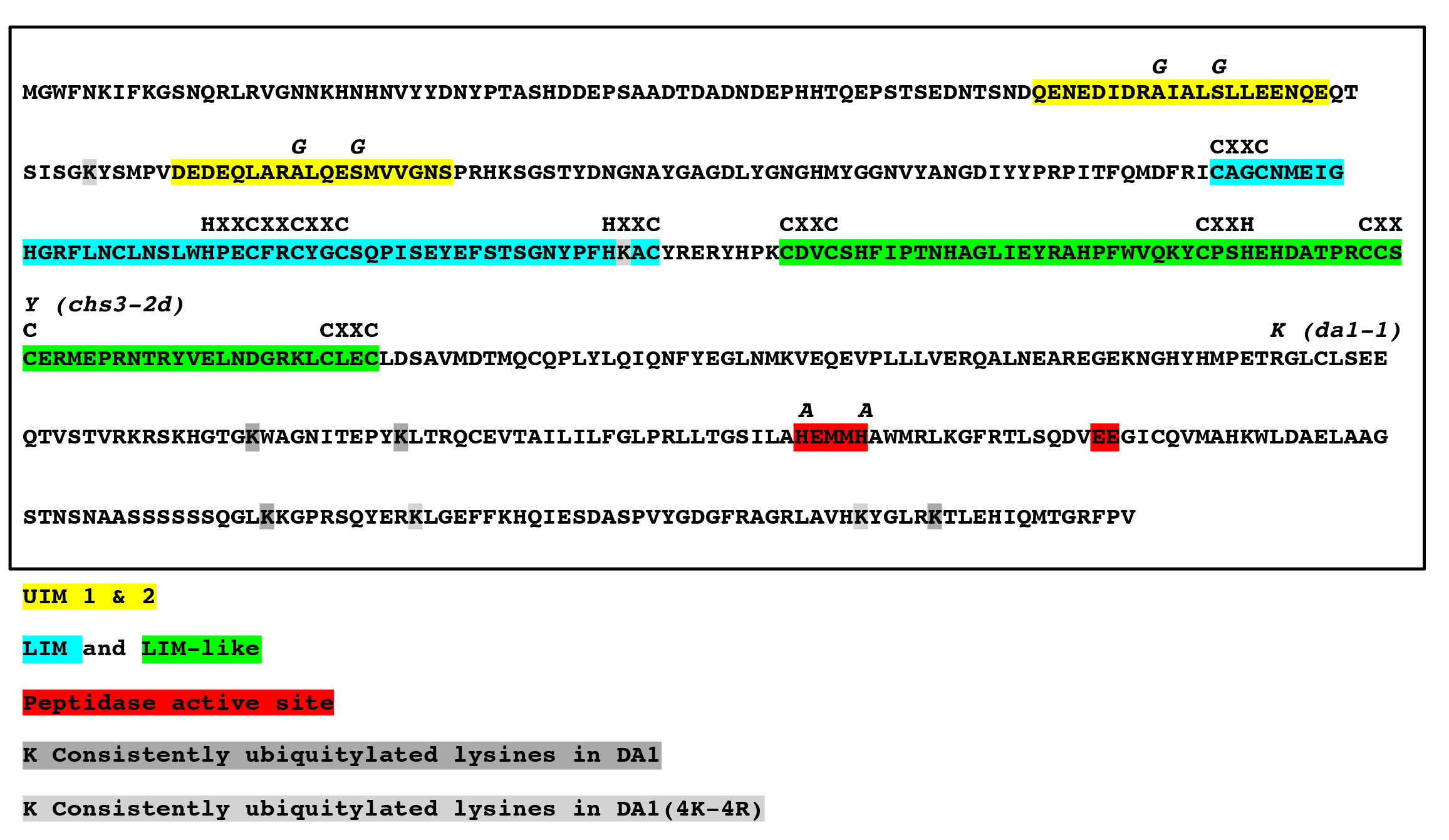


**Supplemental Fig. S4. Diagram summarising DA1 functional domains and positions of ubiquitylated lysines**

The diagram shows the peptide sequence of DA1. Regions with sequence similarity to other proteins with functionally conserved domains are identified. The positions of ubiquitylated lysines in wild-type DA1 and in DA1(4K4R) are highlighted. The location of C and H amino acids predicted to form Zn fingers in the LIM and LIM-like domains is also shown.

**Supplementary Fig. S5. Edman sequencing reactions of cleaved BB-HIS**

This Figure is appended at the end of Supplementary Information

The diagram shows screenshots of HPLC chromatograms from the Edman sequencing reactions of cleaved BB-HIS. The sequencing cycles are shown above each panel and represent the number of residues from the N terminus. DMPTU and DPTU are reaction by-products. The red arrows identify the previously released N-terminal amino acid(s) and the green arrows identify the new N-terminal amino acid(s).


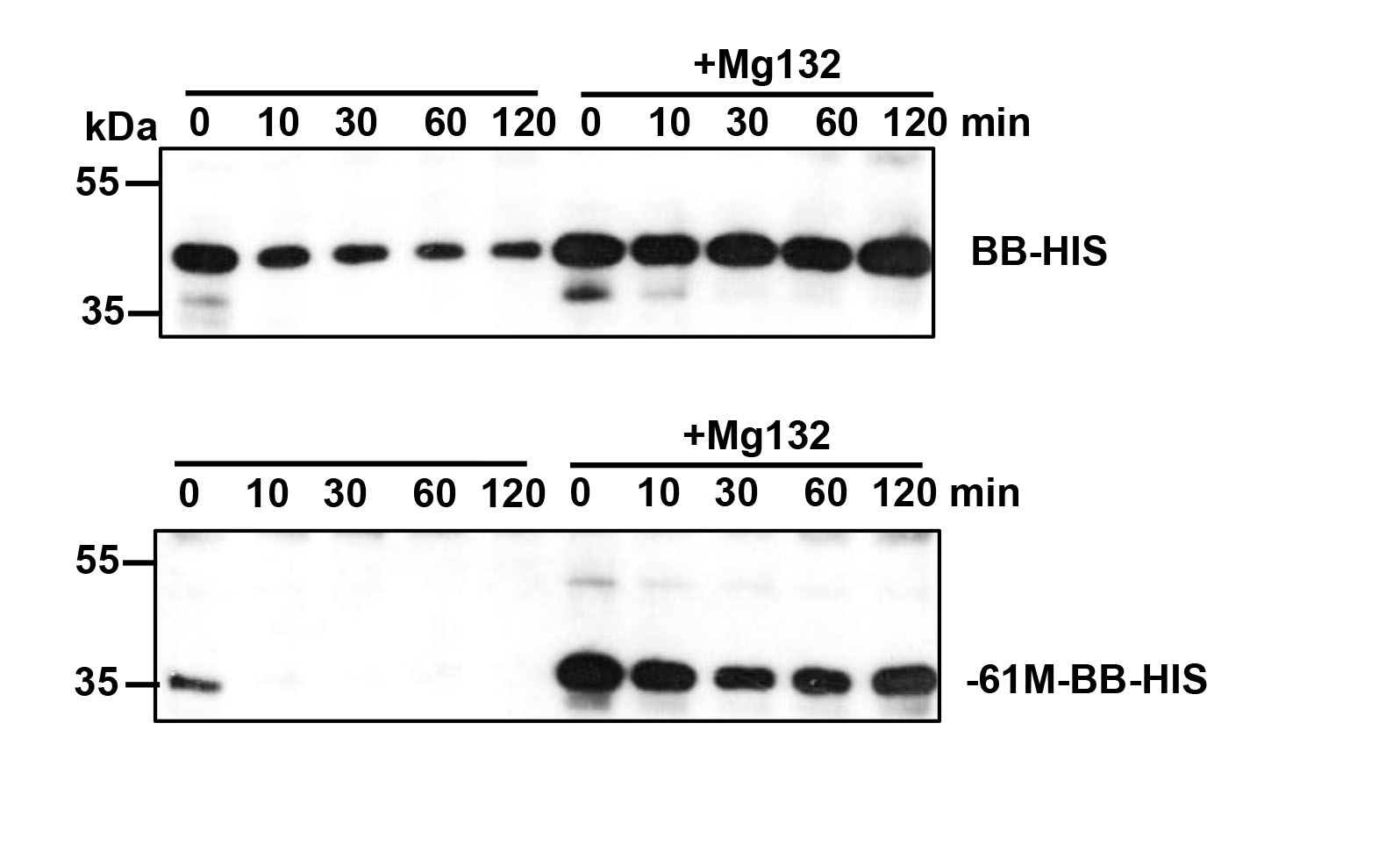


**Supplementary Fig. S6. Cell-free degradation assays of BB and -61M-BB.**

Equal amounts of bacterially- expressed and purified -61M-BB-HIS and BB-HIS proteins were incubated in a cell-free extract of Arabidopsis seedlings and sampled over a 2 h time-course. The immunoblot used anti-HIS to detect -61EOD1-HIS and EOD1-HIS levels in the presence and absence of the proteasome inhibitor MG132.

**Supplementary Fig. S7. Comparison of amino acid sequences in the LIM and LIM-like region of DA1**

Panel A. Clustal O sequence alignment of Arabidopsis DA1 and *Roseiflexus* LIM-peptidase protein sequence showing conserved amino acids in the LIM and LIM-like region of DA1. The positions of identical amino acids are shown by * and structurally related amino acids by : .

Panel B. Diagram of potential LIM and LIM-like Zn finger motifs of DA1 and *Roseiflexus* LIM-peptidase protein sequence. Red dots represent amino acids from *Roseiflexus* and black dots from Arabidopsis DA1. The amino sequence of the region is shown between the diagrams, showing C and H amino acids with the potential to coordinate Zn atoms. The position of C274, which is mutated to Y in the *DAR4 chs3-2d* mutation, is shown.

**Table S1. Primers used for Vector Construction**

| **Primer Name** | **Sequence 5'-3'** |
| --- | --- |
| **Infusion cloning** |  |
| UBP15 infu-1F Xba1 | GACAAGATCTCTAGAATGCTTGAACCAAGGGGAGC |
| UBP15 infu-1R Xba1 | ATTAACTCTCTAGACTACCAGTAACTGTAAGTTC |
| UBP15 infu-2F Xba1 | AGAGGACACGCTCGAGATGCTTGAACCAAGGGGAGC |
| UBP15 infu-2R Xba1 | TATAATCCATCTCGAGCTACCAGTAACTGTAAGTTC |
| TCP5 infu-1F Xho1 | AGAGGACACGCTCGAGATGAGATCAGGAGAATGTGATG |
| TCP5 infu-1R Xho1 | TATAATCCATCTCGAGAGAATCTGATTCATTATCGC |
| TCP5 infu-2F Xba1 | GACAAGATCTCTAGAATGAGATCAGGAGAATGTGATG |
| TCP5 infu-2R Xba1 | ATTAACTCTCTAGATCAAGAATCTGATTCATTATCGC |
| TCP14 infu-2F Xho1 | AGAGGACACGCTCGAGATGCAAAAGCCAACATCAAG |
| TCP14 infu-2R Xho1 | TATAATCCATCTCGAGATCTTGCTGATCCTCCTCATC |
| TCP14 infu-3F Xba1 | GACAAGATCTCTAGAATGCAAAAGCCAACATCAAG |
| TCP14 infu-3R Xba1 | ATTAACTCTCTAGACTAATCTTGCTGATCCTCCT |
| TCP15 infu-1F Xho1 | AGAGGACACGCTCGAGATGGATCCGGATCCGGATCATAAC |
| TCP15 infu-1R Xho1 | TATAATCCATCTCGAGGGAATGATGACTGGTGCTTCCATC |
| TCP15 infu-2F Xba1 | GACAAGATCTCTAGAATGGATCCGGATCCGGATC |
| TCP15 infu-2R Xba1 | ATTAACTCTCTAGACTAGGAATGATGACTGGTGC |
| TCP22 infu-1F Xho1 | AGAGGACACGCTCGAGATGAATCAGAATTCCTCTGTTG |
| TCP22 infu-1R Xho1 | TATAATCCATCTCGAGCTTTTTGTCATCACCACCATTTTC |
| TCP22 infu-2F Xba1 | GACAAGATCTCTAGAATGAATCAGAATTCCTCTGTTG |
| TCP22 infu-2R Xba1 | ATTAACTCTCTAGATCACTTTTTGTCATCACCACC |
| DA1 infu-1F Xba1 | AGATTACGCTTCTAGAATGGGTTGGTTTAACAAGATC |
| DA1 infu-1R Xba1 | TAATTAACTCTCTAGATTAAACCGGGAATCTACCGG |
| **pET-NT cloning** |  |
| DA1-F-BamH1 | GCGGGATCCGGTTGGTTTAACAAGATCTT |
| DA1- (no stop)-R-Xho1 | CGCCGCTCGAGTTAAACCGGGAATCTAC |
| BB-F-BamHI | CGAGGATCCAATGGAGATAATAGACCAGTGGA |
| BB (no stop)-R-XhoI | TATACTCGAGATGAATGCTGGGCTCC |
| BB (stop)-R-XhoI | CCGCTCGAGTCAATGAATGCTGGGCTCC |
| DA2-F-BamHI | TATAGGATCCATGGGTAATAAGTTGGGA |
| DA2(no stop)-R-XhoI | TATACTCGAGTTGCAAGGTAACTTCAGTT |
| BBR-F-EcoRI | TATAGAATTCATGCCCATGGAGAACGACA |
| BBR(no stop)-R-XhoI | TATACTCGAGGCTTTGTCCAGAGGTCGAAG |
| **Topo D/pENTR cloning** |  |
| TOPO-DA1-F | CACCATGGGTTGGTTTAACAAGAT |
| DA1(no stop)-R | AACCGGGAATCTACCGGTCATC |
| DA1(stop)-R | TTAAACCGGGAATCTACCGGTC |
| TOPO-EOD1 fwd | CACCATGAATGGAGATAATAGA |
| BB (stop)-R | TCAATGAATGCTGGGCT |
| BB (no stop)-R | ATGAATGCTGGGCTCC |
| TOPO-DA2 fwd | CACCATGGGTAATAAGTTGGGAAGGA |
| DA2(stop)-R | TTATTGCCAGGTAACTTCAGTT |
| DA2(no stop)-R | TTGCCAGGTAACTTCAGTTG |
| TOPO-BBR fwd | CACCATGCCCATGGAGAACGAC |
| BBR(stop)-R | TCAGCTTTGTCCAGAGGTCGAAGTTGA |
| BBR(no stop)-R | GCTTTGTCCAGAGGTCGAAGTTGA |
| **FLIM-FRET vector construction** |  |
| BB EcoRV fwd | CATGATATCAGAATGGAGATAATAGACCA |
| BB NheI rev | CAAGCTAGCATGAATGCTGGGCTC |
| CACC-GFP-F | CACCATGGCGGCCGCAATGG |
| mRFP-R | TTAGGAATTCGGGGCGCCG |
| **Mutagenic primers** |  |
| *DA1 peptidase active site* |  |
| DA1-GeneArt-HH-AA-F | GGTTCGATTCTAGCTGCAGAGATGATGGCAGCGTGGATGAGGCTC |
| DA1-GeneArt-HH-AA-R | GAGCCTCATCCACGCTGCCATCATCTCTGCAGCTAGAATCGAACC |
| *DA1 C274Y* |  |
| DA1-GeneArt-C-274-Y-F | CGAGATGTTGCAGTTATGAAAGAATGGAGCC |
| DA1-GeneArt-C-274-Y-R | GGCTCCATTCTTTCATAACTGCAACATCTCG |
| *DA1 R358K* |  |
| DA1-GeneArt-R-358-K-F | ACATGCCAGAAACAAAAGGACTCTGCCTTTC |
| DA1-GeneArt-R-358-K-R | GAAAGGCAGAGTCCTTTTGTTTCTGGCATGT |
| *DA1 UIM1 and UIM2* |  |
| UIM1-F | GGATCCGATAATACATCGAAGACCA |
| UIM1-R | CTCGAGTTTCCCGCTTATACTTGTCT |
| OL-F1 | GTGGAATTGCATTGGCGCTTTTAGAAGAGAAT |
| OL-F2 | CGCCAATGCAATTCCACGGTCTATGTCTTCATTTTC |
| UIM2-F | GGATCCAAATACTCGATGCCGGT |
| UIM2-R | CTCGAGTGTACTTCCACTTTTGTGACGG |
| OL-F2 | AGGCCTACAAGAAGGTATGGTAGTTGGGAATTCAC |
| OL-FR | ACCTTCTTGTAGGCCTCTAGCAAGTTGCTCATCTTC |
| **gsGreen-BB cloning** |  |
| gsGreen in-pEARley103 Xho1 F | TTTGGAGAGGACACGCTCGAGATGGTGAGCAAGGGCGAG |
| gsGreen in-pEARley103 Xba1 | TAATTAACTCTCTAGATGAACCGCCTCCACCCGGCTC |
| BB in gsGreen F | AGGCGGTTCAATGAATGGAGATAATAGACCAGTGGAAGATG |
| BB in pEARley103 Xba1 R | GGTCTTAATTAACTCTCTAGATCAATGAATGCTGGGCTCCC |
| **PRT1 cloning** |  |
| ss_prt1_tev | GCTTAGAGAATCTTTATTTTCAGGGGATGGCCGAAACTATGAAAGATATTAC |
| as_prt1_gw | GGGTATCATTCTGTGCTTGATGACTCATTAG |
| **Ubiquitin fusion cloning** |  |
| ss_attB1_UBQ | GGGGACAAGTTTGTACAAAAAAGCAGGCTTAGCCGCCACCATGCAGATCTTCGTCAAG |
| ss_bridge_attB1_UBQ | ACCATGCAGATCTTCGTCAAGACGTTAAC |
| as_UBQ_Y_BB | CTTGTACCCACCTCTAAGTCTTAAGACAAGATGT |
| as_UBQ_G_BB | CTTCCCCCCACCTCTAAGTCTTAAGACAAGATGT |
| ss_UBQ_Y_BB | GTGGGTACAAGTTTGGGTTTTCAGGATCAGAT |
| ss_UBQ_G_BB | GTGGGGGGAAGTTTGGGTTTTCAGGATCAGAT |
| as_BB_HAT | GATAGCCCGCATAGTCAGGAACATCGTATGGGTAATGAATGCTGGGCTCCCCA |
| ss_attB1_M_BB | GGGGACAAGTTTGTACAAAAAAGCAGGCTTAGCCGCCATGGGATACAAGTTTGGGTTTTCAG GATCAGAT |
| ss_HAT_BB | GGGAGCCCAGCATTCATTACCCATACGATGTTC |
| as_HAT_stop | GGGACCACTTTGTACAAGAAAGCTGGGTATCAAGCACCAGCACCAGCGTAATC |
| as_HA_os | GGGGACCACTTTGTACAAGAAAGCTGGGTAAGCACCAGCACCAGCGTAATC |
